# Atomistic Insights into Halide Double Perovskite Nanocrystals obtained by Multistep Synthesis and Efficient Compositional Engineering

**DOI:** 10.1021/acsnano.5c06497

**Published:** 2025-08-11

**Authors:** Nicola Dengo, David F. Macias-Pinilla, Pietro Anzini, Mara Colombo, Simone Virga, Andrea Brambilla, Piero Antonio Zecca, Damiano Monticelli, Francesco Giannici, Federica Bertolotti

**Affiliations:** † Department of Science and High Technology, 19045 University of Insubria, via Valleggio 11, Como 22100, Italy; ‡ Total Scattering Laboratory (To.Sca.Lab), Department of Science and High Technology, 19045 University of Insubria, via Valleggio 11, Como 22100, Italy; § Department of Chemical Sciences, University of Padova, via Marzolo 1, Padova 35131, Italy; ∥ Department of Physics and Chemistry “Emilio Segrè”, 18998 University of Palermo, viale delle Scienze, Ed.17, Palermo 90128, Italy; ⊥ CLIP-Como Lake Institute of Photonics, via Valleggio 11, Como 22100, Italy; # Department of Medicine and Technological Innovation, 19045 University of Insubria, via Guicciardini 9, Varese 21100, Italy

**Keywords:** halide perovskites, total scattering, nanocrystals, density functional theory, photoluminescence, synthesis, solid solutions

## Abstract

Lead-free halide double perovskites (HDP), particularly those within the Cs_2_B^+^InCl_6_ family that show a direct band gap, have recently emerged as promising semiconductors to address key challenges associated with lead-based perovskites, such as toxicity and instability in air and moisture. Their compositional flexibility, structural versatility, and ease of cation transmutation offer considerable potential for bandgap engineering. Here, we present a versatile, multistep, solution-based synthetic strategy for HDP nanocrystals. This method separates the precursor dissolution and reaction stages, providing much greater control over the synthesis. The flexibility of this approach makes it widely generalizable and highly adaptable to high-throughput flow chemistry techniques, including multifluidic platforms and (semi)­automated frameworks (e.g., self-driving or automated laboratories). Here, we demonstrate that stable Cs_2_(Na,Ag)­InCl_6_ and Cs_2_(Na,K)­InCl_6_ HDP compositions can be readily obtained through this approach. Through state-of-the-art atomic-scale experimental and theoretical characterization, we provide insights into the evolution of chemical bonding upon Na^+^/Ag^+^ substitution into the Cs_2_(Na,Ag)­InCl_6_ series. Finally, we investigate the limited miscibility of K^+^ within the NaCl_6_ sublattice of Cs_2_(Na,K)­InCl_6_, which can be ascribed to the distorted pentagonal bipyramidal coordination adopted by K^+^, as observed in the endmember Cs_2_KInCl_6_ composition. All together, these fundamental structural findings serve as a basis for the interpretation of the optical properties of the HDP nanocrystals developed in this work. By combining spectral and structural evidence, we investigate the origins of their absorption and emission properties, with general applicability to similar HDP compositions.

## Graphical abstract

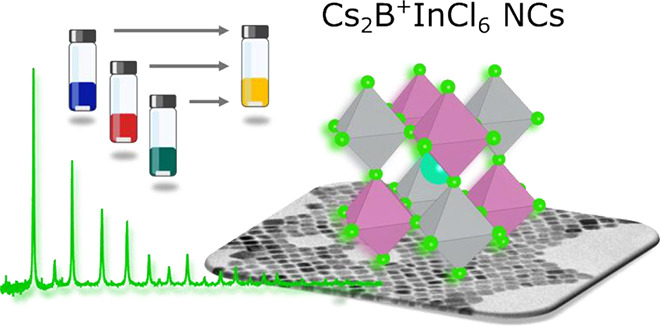

## Introduction

Over the past decade, lead-based halide perovskites have established a milestone in materials chemistry for optoelectronic applications due to their high absorption coefficient, excellent carrier mobility, tunable band gap, and ease of solution processing.^1,2^ However, the toxicity of lead to both humans and the environment, coupled with the poor moisture stability of lead halide perovskites, has inevitably limited practical applications. This has also recently prompted significant efforts to develop alternative lead-free materials with similar optoelectronic properties. A promising strategy to preserve the cubic perovskite structure involves the heterovalent substitution of Pb^2+^ maintaining the average B-site charge, leading to the formation of an alternative 3D “double-perovskite” structure with the general formula A_2_B^+^B^3+^X_6_. While the A^+^ and X^–^ ions remain consistent with the traditional materials (where A^+^ is typically Cs^+^ in all-inorganic and methylammonium in the hybrid perovskites, and X^–^ = Cl^–^, Br^–^, I^–^), the heterovalent replacement of lead opens up a wide array of possible compositions, offering new pathways to thermodynamically stable materials with tailored optoelectronic properties.^3–14^

However, despite some promising results,^15–17^ the optical properties of halide double perovskites (HDPs) still present major challenges for their development and implementation into devices, highlighting the crucial role of synthetic materials chemistry in this field. These challenges arise from intrinsic and surface defects, indirect band gaps, and symmetry-forbidden electronic transitions, all typical of HDPs.^5^ The large number of possible compositions in HDPs has prompted extensive computational studies aimed at streamlining experimental efforts in the search for thermodynamically stable materials with optimal absorption and emission properties, suitable band gaps, high carrier mobility, and enhanced electronic dimensionality.^3,18,19^ It should, however, be noted that despite the large pool of possible compositions predicted to be thermodynamically stable, relatively few have been successfully synthesized so far. On the experimental side, some HDPs have been explored both in bulk form^6,7,12,13^ and as nanocrystals (NCs),^3–5,8^ mostly incorporating alkali metal ions, or Ag^+^, along with In^3+^, Sb^3+^, and Bi^3+^ as trivalent B cations. Structural modulations in HDPs are essential to reduce the band gap and to break the parity forbidden transitions that inherently limit photoluminescence efficiency.^20^ These modulations are typically introduced via doping,^15–17,20–26^ with promising results achieved through homovalent substitution at the In^3+^ site using Bi^3+^ or Sb^3+^. The ns^2^ lone pair electrons of these dopants can promote dynamic symmetry breaking in the excited state, enhancing radiative recombination of the excitons.^27^ For example, in bulk Cs_2_Ag_0.60_Na_0.40_InCl_6_ doped with 4% Bi^3+^, a significant photoluminescence quantum yield (PLQY) of 86% was reported and attributed to the Jahn–Teller distortion of AgCl_6_ octahedra in the excited state. This distortion reduces the inversion symmetry of the electron wave function at the Ag site, thus altering the parity of the self-trapped excitons (STEs) and enabling radiative recombination.^15^ Kovalenko and co-workers achieved a record PLQY of up to 93% and bright blue emission upon doping Cs_2_NaInCl_6_ and Cs_2_KInCl_6_ with Sb^3+^, using single-crystal and powder samples, respectively.^27^

In the case of colloidal NCs, efforts have been made to achieve comparable enhancements starting from essentially nonemissive undoped samples. Liu et al. reported a PLQY of 11.4% for Bi^3+^-doped Cs_2_AgInCl_6_ NCs,^22^ while Zhu et al. achieved a PLQY of ∼15.96% in ultrasmall Cs_2_Ag_0.8_K_0.2_In_0.875_Bi_0.125_Cl_6_ NCs.^28^ The highest PLQY for NCs thus far was achieved by Liu et al., by synthesizing ∼10 nm Bi^3+^-doped Cs_2_Ag_0.20_Na_0.75_InCl_6_ NCs and postsynthetic incorporation of a small amount (7%) of K^+^ ions. This is suggested to induce surface lattice expansion, which improved the ligand passivation efficiency, ultimately resulting in a PLQY of ∼70%.^23^

As demonstrated by these recent studies, although further improvements in PL efficiency are still needed,^29^ due to their single-domain nature, colloidal NCs represent ideal model systems for exploring cationic transmutation in lead-free perovskites and composition-driven bandgap engineering, particularly in relation to their electronic structure and charge transport properties.^30^

Inspired by traditional colloidal syntheses, the usual preparation method for HPD NCs, first reported in 2018, employs the hot-injection one-pot approach.^31–33^ In this method, precursors are dissolved in a high-boiling solvent under heating, stirring, and degassing, followed by the rapid injection of additional reactants to induce nucleation, and a temporally separated NCs growth. While this approach effectively results in NCs with good control over size and shape, it also has important drawbacks, including limited reproducibility due to high operator dependency, infeasibility for large-scale production, and the frequent requirement for inert conditions. Moreover, its compositional tunability (i.e., the key to exploiting HDPs) is limited, since introducing a dopant or adjusting the amount of an alloying cation requires setting up an entirely new reaction.

In this work, we present a multistep synthetic strategy for synthesizing HDP colloids, yielding high-purity (nano)­crystalline phases with exceptional air stability. Unlike conventional one-pot methods, this approach allows rapid and efficient compositional engineering, allowing easy cation substitution within the 3D HDP frameworks, a crucial step toward materials with tailored optoelectronic properties. This strategy is first demonstrated here on well-established HDP compositions with the general formula Cs_2_(Na,Ag)­InCl_6_, and then extended to the Cs_2_(Na,K)­InCl_6_ system, which has thus far been only superficially explored. By integrating a thorough structural analysis from high-resolution synchrotron X-ray total scattering data obtained from newly synthesized HDP colloids with ab initio Density Functional Theory (DFT) calculations, we provide insights at the atomic level into chemical bonding, band structures, and structural stability in response to compositional changes in HDP NCs. These fundamental findings are ultimately used to provide a comprehensive interpretation of the optical spectral features of Cs_2_B^+^InCl_6_ (where B^+^ = Na^+^, Ag^+^, K^+^), as well as the origin of their emission properties arising from the mixed (Na,Ag) compositions. This is made possible by the easy availability of a wide range of HDP stoichiometries in the form of stable NCs, allowed by the original synthetic approach developed in this work.

## Results and Discussion

### Multistep synthesis of Cs_2_B^+^InCl_6_ nanocrystals

The synthetic approach developed in this work for synthesizing direct band gap Cs_2_B^+^InCl_6_ (where B^+^ = Na^+^, Ag^+^, K^+^) NCs is summarized in Figure 1a,b. A *base solvent* (*BSv*) is prepared as a common medium for dissolving all the precursor stock solutions by mixing 1-octadecene (ODE, 100 mL), oleylamine (OAm, 6.6 mL), and oleic acid (OA, 25 mL). The mixture is stirred, heated in an oil bath at 140 °C, and degassed under vacuum for 20 min, resulting in a light-yellow solution. Precursor stock solutions for the target elements are prepared in separate vials (Figure 1a) by dissolving the corresponding precursors [Cs_2_CO_3_, In­(OAc)_2_OH, Na­(OAc), K­(OAc)] into the *BSv* in an oil bath at 140 °C and continuous stirring until dissolution (typically within 1 h; Figure 1a, full details in the Methods and Supporting Information). The Ag-precursor stock solution is prepared by dissolving the Ag­(OAc) into the *BSv* with the addition of triphenylphosphine (to promote Ag­(OAc) solubility), followed by gentle heating in a 70 °C oil bath to ensure complete dissolution. This relatively low temperature, optimized through preliminary tests, is specifically used to prevent the reduction of Ag­(I) by OAm.^23,34^ The nucleation of metallic Ag particles, which are detrimental to the PL quantum yield,^23^ can be easily detected as the solution turns deeply dark. The presence of the triphenylphosphine allows the full dissolution to occur while keeping the low temperature necessary to avoid the reduction to Ag(0) in the process, as further supported by the spectroscopic investigation presented below. The precursor stock solutions can be stored in air and at room temperature and are employed as cation reservoirs for exploring various molar ratios in B^+^-site alloying and doping. To synthesize the target compositions [Cs_2_(Na,Ag)­Cl_6_ and Cs_2_(Na,K)­Cl_6_], the required volumes of each stock solution are mixed in a septum-sealed vial. This vial is heated to 170 °C for 220 s while 150 μL of the preheated BzCl stock solution is injected (Figure 1b). The mixture turns white and opaque, and, after 10 s, the reaction is quenched by rapid cooling in an ice–water bath. The resulting precipitate is purified and extracted with toluene, yielding cuboidal NCs with an average edge length of ∼30 to 40 nm for Cs_2_(Na,Ag)­Cl_6_ (Figures 1c and S1), and fairy larger (>100 nm) for the Cs_2_KInCl_6_ endmember. The relatively larger size of NCs obtained with the developed multistep approach, compared to the conventional one-pot methods (yielding ∼10 to 20 nm NCs), does not present a significant drawback, as there is limited literature evidence suggesting distinctive optoelectronic properties arising from quantum confinement at ultrasmall sizes (<20 nm) in HDP NCs.^35,36^ This is further supported by the spectroscopic analysis presented in this work. Figure 1d showcases high-resolution TEM (HRTEM) analysis performed on Cs_2_Na_0.62_Ag_0.38_InCl_6_ NCs, confirming the formation of Ag(0) nanoparticles upon electron beam exposure.^30,34,37^ This conclusion is supported by the interplanar distances of the two components (blue square: HDP; red ellipse: dark spots observed on the NCs in Figure 1c), and by the relative Fast Fourier Transform (FFT). Furthermore, the presence of additional undetected secondary phases in the synthesized samples is ruled out by energy-dispersive X-ray spectroscopy (EDX, Figure S2), showing a homogeneous distribution of Cs, Na, Ag, In, and Cl across all the scanned particles.

**Figure 1:**
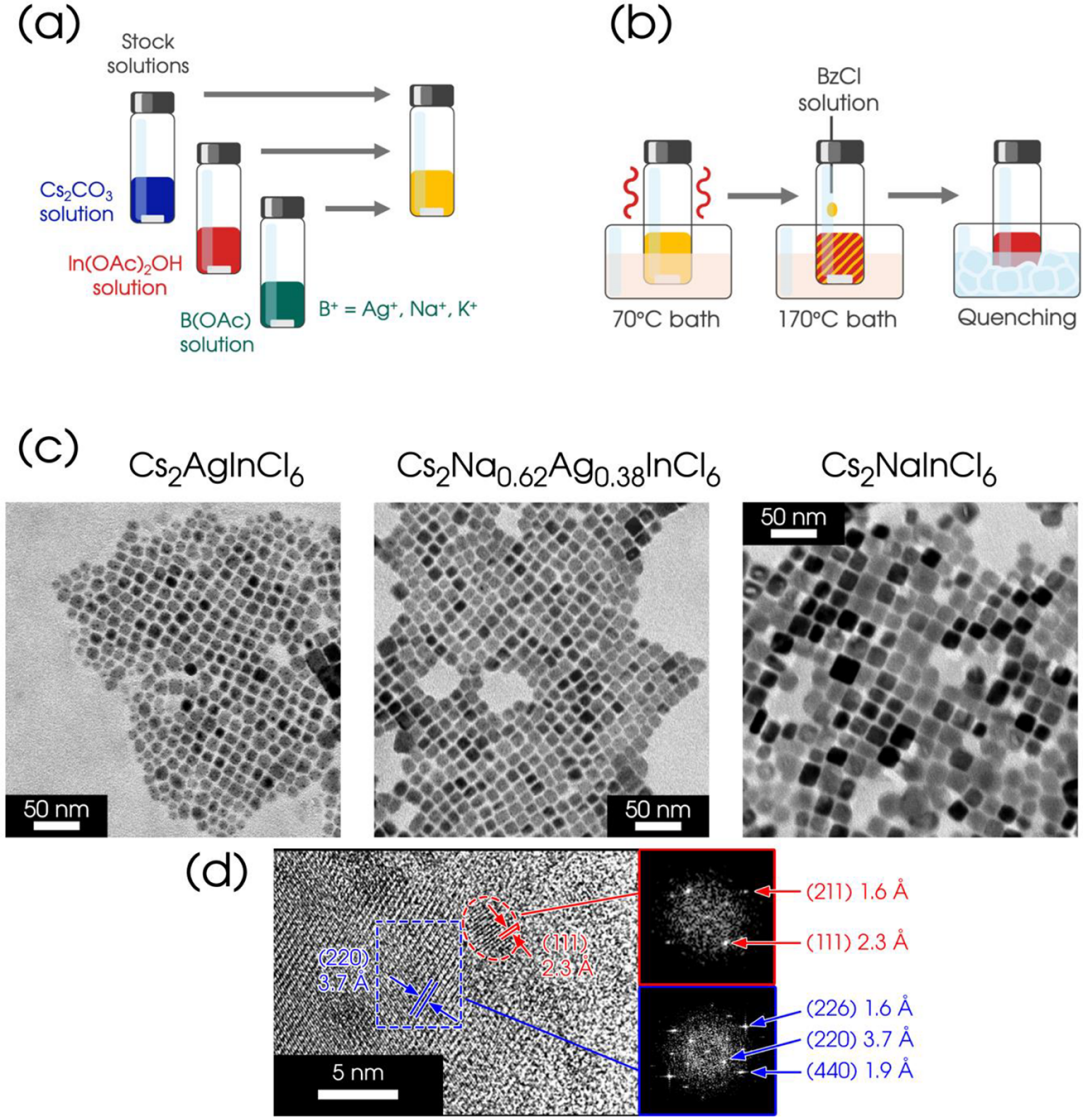
(a, b) Multistep synthesis scheme for Cs_2_B^+^InCl_6_ NCs. (c) TEM images of Cs_2_(Na,Ag)­InCl_6_ NCs, with a scale bar of 50 nm. The dark spots on Cs_2_(Na,Ag)­InCl_6_ NCs are ultrasmall (∼3 to 4 nm) metallic nanoparticles forming under TEM,^30,34,37^ as much as it is observed for the lead counterpart.^38^ (d) HRTEM images of the Cs_2_Na_0.62_Ag_0.38_InCl_6_ sample, showing a large NC containing smaller dark spots attributed to the formation of Ag(0) nanoparticles under the electron beam. The blue square and the corresponding FFT images (right) highlight the interplanar distances associated with the HDP (see structural details below). The red ellipse marks one of the dark spot observed in the Ag-containing HDPs in panel (c); the corresponding FFT image on the right displays the main spots attributed to Ag(0) crystal structure (*a* = 4.0855(2) Å),^39^ along with their characteristic interplanar distances.

The synthesized colloidal suspensions of NCs are typically stored in anhydrous solvents under air-free conditions; however, samples stored in air remain stable for at least several weeks (Figure S3). Elemental analysis by ICP-MS, summarized in Table S1, confirms that the HDP NC stoichiometries are in line with the following X-ray scattering characterization, also considering the fundamentally different nature of the sample analyzed (solubilized ions vs solid, (nano)­crystalline samples) and the different sensitivities of the two techniques. The largest deviations are indeed observed for the sample with mixed (Na,Ag) composition Na_0.34_Ag_0.66_ (Na_0.59_Ag_0.65_ from ICP-MS) and can be further rationalized in light of its peculiar structural features revealed by X-ray scattering, as detailed in the next paragraph.

All attempts to synthesize bromide analogs of the proposed HDP NCs with benzoyl bromide as halide precursor were unsuccessful. This aligns with previous reports indicating that Cs_2_AgInBr_6_, even in the bulk form, is thermodynamically unstable at room temperature, despite favorable Goldschmidt (0.93) and octahedral (0.50) tolerance factors (although the latter becomes unfavorable, i.e. <0.414, if only InBr_6_ octahedra are considered, being In^3+^ too small to coordinate six bromides^40^). To date, only two experimental reports have claimed the synthesis of this material: one via a mechanochemical route, where the resulting product was unstable under illumination,^41^ and another employing thermal evaporation of CsBr, AgBr, and InBr_3_ to form thin films, which rapidly decomposed within 1–2 h into secondary phases such as CsBr, AgBr, and InBr_3_.^42^ On the theoretical side, predictions regarding the thermodynamic stability of Cs_2_AgInBr_6_ remain controversial. While several studies supported its stability based on formation energies and decomposition enthalpies relative to ternary and binary bromide phases,^43–48^ other reports indicate that Cs_2_AgInBr_6_ may be metastable or unstable, with decomposition pathways favoring the formation of Cs_2_AgBr_3_, Cs_2_In_2_Br_9_, and AgBr.^19,49,50^ Furthermore, phonon dispersion calculations indicate dynamical instability, as evidenced by imaginary modes in the vibrational spectrum.^51^

To clarify these conflicting findings, we performed DFT calculations to assess the decomposition energetics of Cs_2_(Na,Ag,K)­Br_6_ compositions, as shown in Figure S4 and Table S2 of the Supporting Information. Our results reveal negative decomposition enthalpies, indicating spontaneous degradation into Cs_2_AgBr_3_ (for Ag^+^ containing HDP), and Cs_2_In_2_Br_9_ + B^+^Br (binary bromides). These results support the hypothesis of intrinsic thermodynamic instability in the bromide analogs of these HDPs.

### Atomic-scale Insights into Cs_2_B^+^InCl_6_ (with B^+^ = Na^+^ Ag^+^) Nanocrystals

We first examine the structural evolution of the Cs_2_Na_*x*_Ag_1–*x*_InCl_6_ HDP (where 0 ≤ *x* ≤ 1), a direct bandgap semiconductor that, when alloyed with Bi^3+^, displays a record PL efficiency of 86% in bulk^15^ and 31.1% in NC form.^36^ These exceptional performances have been attributed to the Jahn–Teller distortion of the BCl_6_ octahedra in their excited state, induced by Na^+^/Ag^+^ alloying, resulting in a bright yellow emission that is enhanced compared to the endmembers (Cs_2_NaInCl_6_ or Cs_2_AgInCl_6_).^36^ Dipole transition element calculations presented in Figure S5 show an enhanced transition probability in the Γ-X path for the mixed Na^+^/Ag^+^ compositions compared with the endmembers, further supporting these literature observations.^15,17,52^

To investigate potential structural distortions occurring upon Na^+^/Ag^+^ alloying even in the equilibrium structures of Cs_2_(Na,Ag)­InCl_6_ NCs, we collected and analyzed high-resolution synchrotron wide-angle X-ray total scattering (WAXTS) data directly from the synthesized colloidal suspensions. Details of the data collection and the thorough data reduction procedure used to extract reliable structural parameters from WAXTS data are provided in the Methods and Supporting Information file. Given the relatively large size of HDP NCs synthesized with the proposed approach, we employed conventional Rietveld refinement to extract structural and microstructural parameters from the experimental data (synoptically reported in Table S3), rather than relying on computationally expensive approaches based on the Debye Scattering Equation,^53–55^ which we have developed and extensively applied to ultrasmall lead halide perovskite NCs.^56–58^

The solvent-subtracted synchrotron WAXTS data of Cs_2_Na_*x*_Ag_1–*x*_InCl_6_ (where 0 ≤ *x* ≤ 1) NCs are shown in Figure 2a for selected stoichiometries. As observed in other studies, all samples crystallize in the archetypal *Fm3̅m elpasolite* structure, consistent with the bulk materials (ICSD entries 257115 and 161183),^40,59^ without any detectable secondary phases. The successful Na^+^/Ag^+^ alloying is qualitatively witnessed by the increase in the intensity of the 111 (*Q* ∼ 1.0 Å^–1^) peak (marked with a red arrow in Figure 2a), reflecting the substitution of Ag^+^ with Na^+^ in the B^+^Cl_6_ octahedra. Rietveld refinement of the *elpasolite* structure against the experimental WAXTS data (Figure S6), reveals that the increase in Na^+^ content induces a small but detectable lattice expansion, from 10.4858(2) Å for Cs_2_AgInCl_6_ to 10.53188(5) Å for Cs_2_NaInCl_6_ NCs (Δ*a/a* = 0.44%). The relative lattice expansion compared to the bulk^40,59^ is more pronounced for Cs_2_AgInCl_6_ (0.18%) than for Cs_2_NaInCl_6_, which shows a negligible change instead (−0.02%). This difference is likely due to the smaller particle size of the former compared to the larger Cs_2_NaInCl_6_ sample analyzed (Table S3), resulting in a surface-induced lattice expansion, a phenomenon commonly observed in many ionic compounds,^60^ including lead-based halide perovskites.^58^ Figure 2b shows that the refined lattice parameters of Cs_2_Na_*x*_Ag_1–*x*_InCl_6_ NCs, with comparable (nano)­crystal sizes (*x* < 1), follow a linear trend against Na^+^ fraction (as refined from the synchrotron WAXTS data, Table S3), following the Vegard’s law prediction for ideal solid solutions. Small deviations from this trend are observed at the lowest Na^+^ fraction [*x*(Na^+^) = 0.34], likely due to local clustering of Na^+^ cations either inside the NCs or at the surface, as suggested for the K^+^ doping of Cs_2_Na_*x*_Ag_1–*x*_InCl_6_ NCs.^23^ This observation is further supported by the broad low-angle shoulder observed for all diffraction peaks for this composition (Figure S7). On the other hand, as the Na^+^ fraction increases, the linear trend of the lattice parameters with Na^+^/Ag^+^ alloying is restored, along with the ideal peak profiles across all scattering angles.

**Figure 2:**
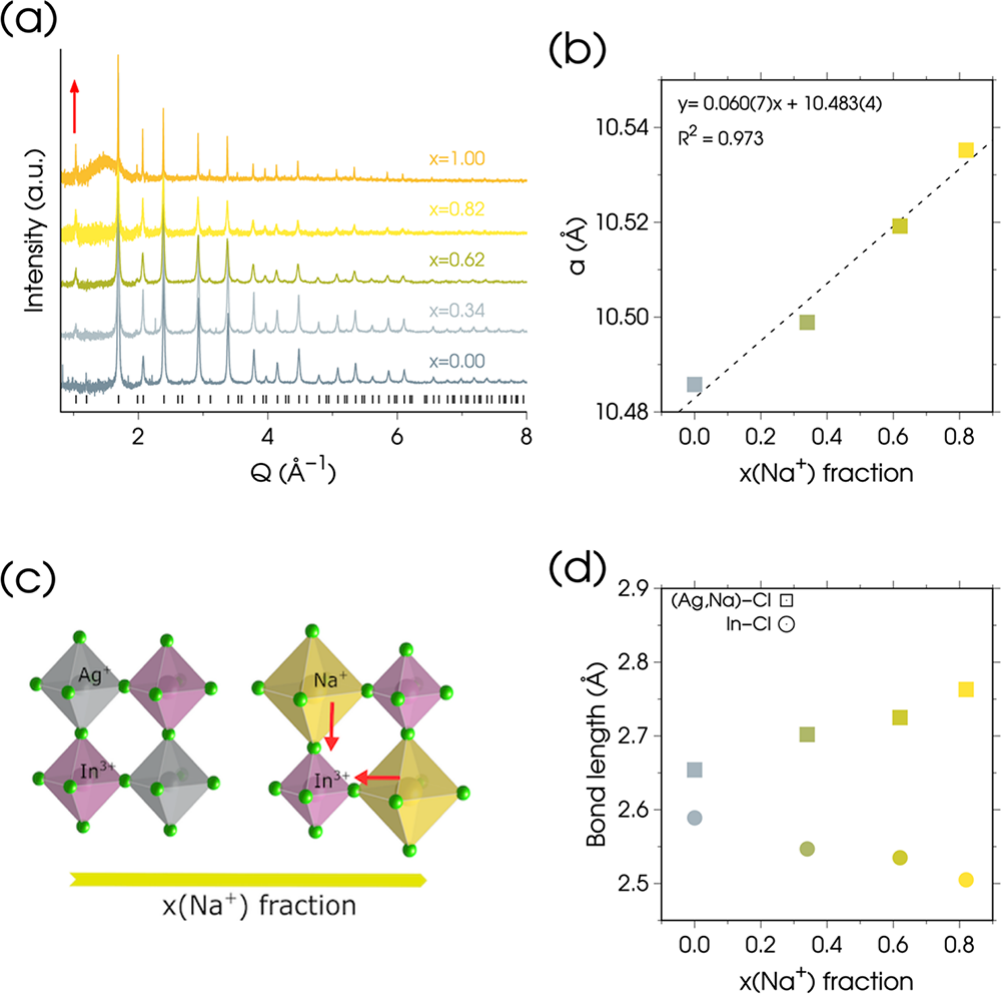
(a) High-resolution synchrotron WAXTS data of Cs_2_(Na,Ag)­InCl_6_ colloidal NCs, with the toluene subtracted for clarity. The red arrow highlights the intensity evolution of the 111 peak upon Na^+^/Ag^+^ substitution. (b) Refined unit cell parameter (*a,* Å) of the *elpasolite* structure as a function of Na^+^ content, with a linear fit according to Vegard’s law for ideal solid solutions. (c) Schematic representation of the deformation of (Na,Ag)­Cl_6_ and InCl_6_ octahedra upon Na^+^/Ag^+^ alloying, derived from the structural analysis of WAXTS data reported in (a) (the deformations are exaggerated for clarity). (d) Evolution of (Na,Ag)-Cl (squares) and In–Cl (circles) bond distances as a function of the Na^+^ fraction (*x*), supporting the structural trends depicted in (c). In (b) and (d) the uncertainties on the unit cell parameters and bond lengths are smaller than the markers.

The lattice expansion from Cs_2_AgInCl_6_ to Cs_2_NaInCl_6_, already reported recently,^15^ is also supported by ab initio DFT geometry optimization (Table S4). However, it is somewhat unexpected when considering the tabulated ionic radii of hexacoordinated Ag^+^ (1.15 Å) and Na^+^ (1.02 Å).^61^ Additionally, in Figure 2c,d, we analyze the evolution of (Na,Ag)-Cl (*d*_Na/Ag–Cl_) and In–Cl (*d*_In–Cl_) bond distances, refined from high-resolution WAXTS data, as Na^+^ fraction (*x*) increases. In the Cs_2_AgInCl_6_ structure [*x*(Na^+^) = 0], the two bond lengths are closer (*d*_Ag–Cl_ = 2.65(1) Å and *d*_In–Cl_ = 2.59(1) Å, Δ*d* = 0.06 Å). As Na^+^ replaces Ag^+^, these two distances diverge, reaching *d*_Na/Ag–Cl_ = 2.763(6) Å and *d*_In–Cl_ = 2.505(6) Å, Δ*d* = 0.258 Å at *x*(Na^+^) = 0.82.

Taken together, all these findings suggest that the Cs_2_(Na,Ag)­InCl_6_ structure not only expands isotropically upon Na^+^/Ag^+^ alloying (Δ*a*/*a* = 0.44%) but also undergoes a progressive deformation of the (Na,Ag)­Cl_6_ and InCl_6_ octahedra: as the latter shrink (Δ*d*/*d* = −3.28%), the former expand (Δ*d*/*d* = 4.26%), so the chlorides are pulled toward In^3+^ while Ag^+^ is gradually replaced by Na^+^, as shown in Figure 2c. This observation is again quite counterintuitive from ionic radii alone (since *r*_Ag_^+^ > *r*_Na_^+^), which are typically used to predict and rationalize the structures of both lead-based and lead-free halide perovskite.^61–63^

To explain these trends, we compare the calculated electron density of these systems in Figure 3a, focusing on the nature of the (Na,Ag)-Cl and In–Cl chemical bonds. Atomic boundaries are identified as the electron density minima between atomic pairs, offering insight into the bonding features. The nature of the In–Cl chemical bonds remains almost unperturbed across all Na^+^/Ag^+^ stoichiometries investigated, and it shows characteristics more akin to the Ag–Cl bond in terms of electron density distribution between the two atomic basins. In contrast, as expected from the larger electronegativity difference between Na and Cl (Δχ_Na–Cl_ = 2.23) compared to Ag and Cl (Δχ_Ag–Cl_ = 1.23), the Na–Cl bond shows a more pronounced ionic character (Figure 3a). Therefore, the shorter B^+^–Cl bond distance experimentally observed in Cs_2_AgInCl_6_ HDPs, which progressively expands upon Na^+^/Ag^+^ substitution, can eventually be attributed to the greater covalent character of the Ag–Cl bond compared to the Na–Cl bond. This feature is also observed in In–Cl, and it helps to explain the greater similarity in the sizes of the AgCl_6_ and InCl_6_ octahedra, in contrast to the progressive structural modification upon the replacement of Ag^+^ by Na^+^.

**Figure 3:**
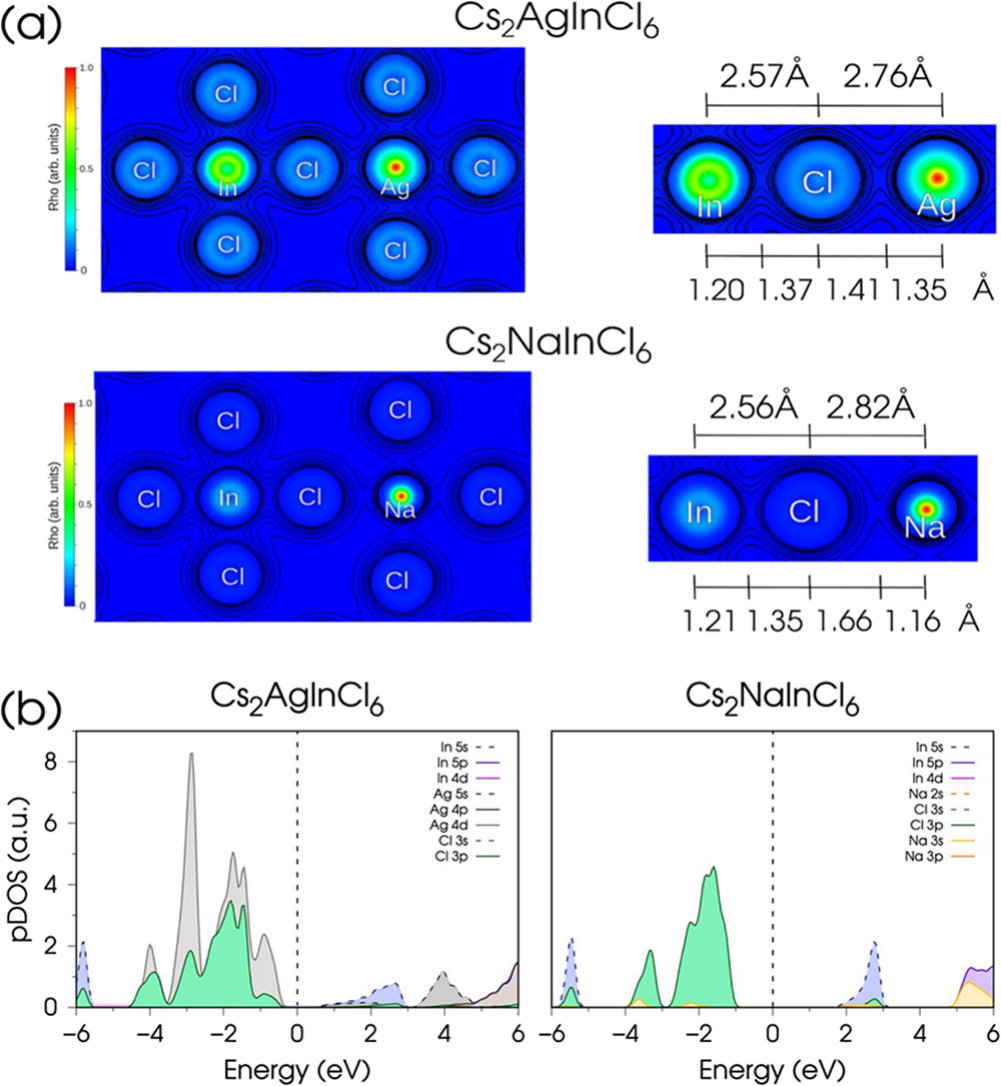
(a) Ground state electronic charge density maps on the (100) plane of Cs_2_AgInCl_6_ and Cs_2_NaInCl_6_, from DFT optimized structures, and (b) pDOS of Cs_2_AgInCl_6_ and Cs_2_NaInCl_6_ with the different atomic contributions close to the band gap, aligned to Cs 5s maximum. Contributions to pDOS from Cs are omitted for clarity.

This finding is further supported by the analysis of the projected Density of States (pDOS, aligned to Cs 5s states) shown in Figure 3b. The minimum of the conduction band (CBM) in Cs_2_NaInCl_6_ is mainly composed of In 5s states, while the valence band maximum (VBM) is predominantly formed by Cl 3p states.^17^ The overlap between Na 3p and Cl 3p states is minimal, which is consistent with a clearly ionic Na–Cl bond. On the other hand, the CBM of Cs_2_AgInCl_6_ is mainly composed of Cl 3p orbitals and In 5s/Ag 5s states, while the VBM is formed by Cl 3p and In 4d/Ag 4d states. The contribution of Ag 4d states close to the band gap indicates a significant covalent character of the Ag–Cl bond compared to Na–Cl.

### Investigating the K^+^/Na^+^ Substitution in Cs_2_B^+^InCl_6_ Nanocrystals

Inspired by the promising recent reports on K^+^ incorporation within the Cs_2_B^+^InCl_6_ lattice, resulting in enhanced optical performance,^23^ we further investigate the general applicability of the synthetic method developed by exploring the K^+^/Na^+^ substitution. Here, we successfully incorporate K^+^ in the Cs_2_NaInCl_6_ lattice up to 22%, still resulting in the archetypal *elpasolite* structure. This incorporation leads to a progressive lattice expansion, from 10.53188(5) Å for Cs_2_NaInCl_6_ to 10.569(2) Å for Cs_2_Na_0.78_K_0.22_InCl_6_ (Figure S8). The dipole transition element calculations shown in Figure S9 further indicate enhanced probability for direct transitions in the Γ-L path for the mixed Cs_2_Na_0.5_K_0.5_Cl_6_ composition compared to the endmembers, consistent with the trend observed for Na^+^/Ag^+^ alloying.

All attempts to synthesize Cs_2_Na_*x*_K_1–*x*_InCl_6_ NCs, with *x* < 0.78 were unsuccessful, resulting in the concurrent precipitation of a hydrated 0D perovskite phase (Figure S8).^27^ This occurred despite the still favorable tolerance factors, ranging from 0.92 (Cs_2_NaInCl_6_) to 0.87 (Cs_2_KInCl_6_), and satisfying both the structural stability criterion for HDPs (*t* < 0.84 for chlorides),^63^ and their positive decomposition enthalpies reported in Figure S4. To further elucidate the partial immiscibility of K^+^ in the Cs_2_NaInCl_6_ 3D perovskite structure, we analyze the coordination environment of the different cations with chlorides. According to Paulings’ first rule, the so-called octahedral factor, μ = r_B_/r_X_, is nearly ideal for InCl_6_ (0.441), followed by NaCl_6_ (0.564) and KCl_6_ (0.762). The higher μ value computed for KCl_6_ suggests that K^+^, being significantly larger than In^3+^ or Na^+^, may favor adopting a higher coordination number than six, potentially leading to different coordination polyhedra. Figures S10 and 4a,b show high-resolution synchrotron WAXTS data for Cs_2_KInCl_6_ in colloidal suspension (toluene) and dried forms, respectively, characterized by significant air and structural stability (Figure S3). The high-resolution data set from the colloidal suspension reveals a systematic splitting of the main reflections (in particular, the 220, 400, 422, and 440 in cubic notation, observed at *Q* values around 1.6, 2.3, 2.9, and 3.3 Å^–1^ respectively), highlighted by red arrows in Figure S10. This finding is clearly in disagreement with the expected *elpasolite* crystal structure that is indeed observed for Cs_2_Na_*x*_K_1–*x*_InCl_6_ compositions with *x*(Na^+^) ≥ 0.78.

**Figure 4:**
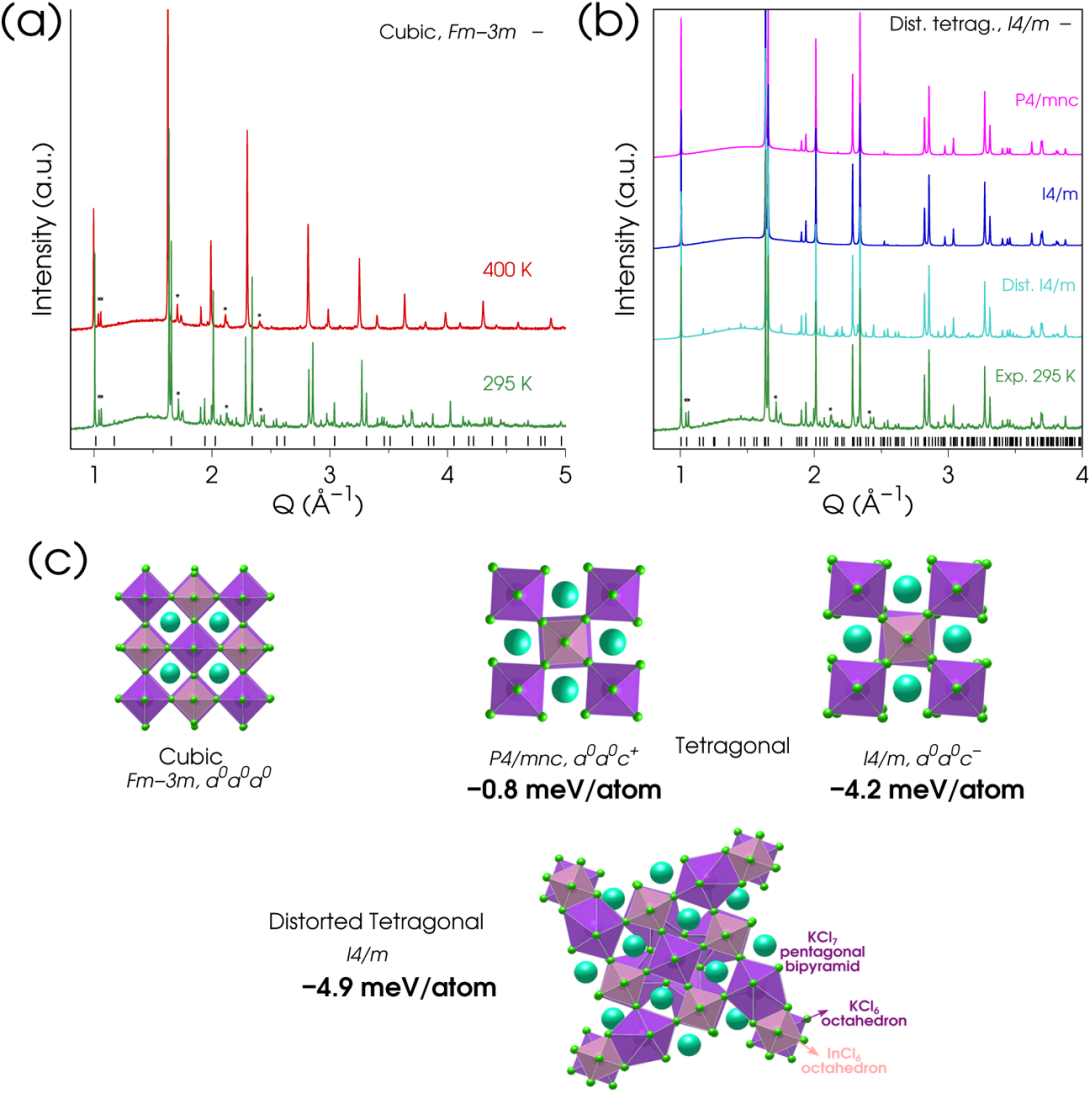
(a) Variable temperature high-resolution X-ray TS data collected on dried Cs_2_KInCl_6_ sample. Data from the corresponding colloidal suspension in toluene at RT are reported in Figure S10. A minor impurity phase, precipitated upon drying, is present in both the RT and 400 K data sets and is marked with asterisks in (a) and (b). (b) Experimental WAXTS data (green trace) collected at RT on Cs_2_KInCl_6_ compared with simulations performed using the three tetragonal crystal structures shown in (c). From bottom to top, the distorted *I4/m* structure (light blue curve) and the two “regular” tetragonal *I4/m* (blue trace) and *P4/mnc* (magenta trace) phases. (c) Refined cubic and tetragonal structures derived from Cs_2_KInCl_6_ WAXTS data, with their corresponding Glazer notation indicating octahedral rotation modes: in-phase (+) and antiphase (−) cooperative rotations of subsequent layers, with reference to the cubic structure (zero tilt, 0). The total energy for each phase calculated by DFT, relative to the cubic phase, is also reported in bold.

Starting from the isostructural compounds CsInCl_3_ ^64^ and the unit cell parameters reported by Kovalenko and co-workers,^27^ we refined the crystal structure of Cs_2_KInCl_6_ as a tetragonal HDP (space group *I4/m*) with anomalous connectivity and unit cell parameters (*a* = *b* = 16.97349(3) Å, *c* = 10.99503(3) Å) using Cs_2_KInCl_6_ data measured under dry conditions to improve the counting statistics (Figures 4a and S11). The optimized atomic coordinates and atomic displacement parameters are reported in Table S3 and in the deposited Crystallographic Information File (CSD 2433763). Notably, upon heating the sample to 400 K, the archetypal *Fm3̅m* cubic structure characteristic of the other end members of the HDP family is restored (Figures 4a and S11). The distorted tetragonal crystal structure of Cs_2_KInCl_6_ obtained at room temperature (RT) does not align with the expected symmetry reduction from the *Fm3̅m elpasolite* structure, according to the group-subgroup relationships.^65^ The two tetragonal structures resulting from the cooperative rotations of the KCl_6_ and InCl_6_ octahedra are shown in Figure 4c, along with the corresponding Glazer notation. The simulations presented in Figures 4b and S11 demonstrate that, although the main peaks are common across all tetragonal phases, only the distorted *I4/m* structure accounts for most of the superstructure peaks found in the high-resolution WAXTS data. Starting from the experimental structural models, we performed DFT geometry optimizations, and the calculated total energies relative to the cubic model of the corresponding structures are compared in Figure 4c (in bold). While the energy differences between the various crystal symmetries are within 5 meV/atom (i.e., comparable with the thermal energy at room temperature), all three tetragonal Cs_2_KInCl_6_ models (detailed in Table S5) are found to be more stable than the cubic *elpasolite* phase, with the distorted *I4/m* structure being the most energetically favorable, in line with the experimental findings. The analysis of the atomic connectivity (model in Figures 4c and S12) reveals the octahedral coordination of all In^3+^ (with an average In–Cl bond distance of 2.51(4) Å), 1/5 of which are rotated by 45° in the *ab*-plane along their 4-fold octahedra axes, and the same octahedral coordination for 1/5 of K^+^ (average K–Cl 2.95(2) Å) cations. The remaining K^+^ cations adopt a distorted pentagonal bipyramidal coordination with an average K–Cl bond length of 3.03(5) Å and undergo a large displacement of ∼0.84 Å from their ideal K^+^Cl_6_ position toward the rotated InCl_6_ octahedron. This distorted seven- (and even 7 + 1)-fold coordination has been observed for K^+^ in other HDP bulk structures,^66–69^ and attributed to the large ionic radius difference between the K^+^ and B^3+^ cations (Δ*r* = *r*_K^+^_ – *r*_In^3+^_ = 0.58 Å in Cs_2_KInCl_6_ NCs) coupled with a tilting instability of the B^+^X_6_ octahedra (*t* in the 0.89–0.93 range). Under these conditions, the smaller B^3+^X_6_ octahedra undergo localized (noncooperative) rotations (sometimes as large as 45°, as observed for Cs_2_KInCl_6_ NCs), which disrupt the corner-sharing connectivity between B^+^ and B^3+^ and replace it with edge-sharing polyhedra.

A significant volume expansion occurs when transitioning from the RT tetragonal structure to the *elpasolite* structure at 400 K, with a lattice parameter increase of 1.008%.[Fn fn1] The expansion results mainly in bond angle relaxations rather than in bond distance modifications: in the refined crystal structure at 400 K, the In–Cl and K–Cl bonds become 2.484(1), and 2.981(1) Å, respectively. This trend is also consistent with the gradual contraction of the InCl_6_ octahedra while the B^+^Cl_6_ octahedra expand moving from Cs_2_NaInCl_6_ to Cs_2_KInCl_6_, driven by the larger B^+^ cationic size (*r*_Na+_ = 1.02 Å; *r*_K+_ = 1.38 Å).^61^

### Impact of Atomic Structure on the Optical Properties of Cs_2_(Na,Ag)­InCl_6_ and Cs_2_(Na,K)­InCl_6_ Nanocrystals

To complete our atomic-level understanding of Cs_2_B^+^InCl_6_ (with B^+^ = Ag^+^, Na^+^, K^+^) NCs, we present here a correlation between their detailed structural characterization and optical properties, most of which remain unclear in recent literature. The HDPs presented here are all direct band gap semiconductors with parity-forbidden transitions between the band edges, as both the CBM and VBM share the same even parity, with some compositional variations. Cs_2_NaInCl_6_ and Cs_2_KInCl_6_ are characterized by parity-forbidden transitions between the band edges in the whole reciprocal space, while Cs_2_AgInCl_6_ has parity-forbidden transitions only between CBM and VBM, and allowed transitions at other k points.^70^ These computational results have been experimentally supported, for example, for bulk Cs_2_AgInCl_6_, where a strong absorption feature is observed below 300 nm (>4.1 eV),^40,71,72^ followed by a weaker peak at approximately 378 nm (∼3.3 eV), attributed to an allowed transition and the parity-forbidden transition, respectively.^6,35^

For HDP NCs, the evolution of the absorption spectra as a function of Na^+^/Ag^+^ alloying has been reported recently,^36^ highlighting a sharp band-edge exciton peak at 269 nm for Ag-doped Cs_2_NaInCl_6_ NCs, which exhibit enhanced PL properties. In Figures 5a–c and S13 we report the optical UV–vis absorption and emission spectra of various Cs_2_B^+^InCl_6_ compositions, easily accessible through the multistep approach developed in this work, including both endmembers and mixed stoichiometries. Surprisingly, we found that the sharp peak of the absorption spectra highlighted in ref. 36 (Figure S13) appears at exactly the same wavelength (269 nm) for all HDP NCs, regardless of the B^+^ cation occupying the perovskite framework. If one were to follow the literature interpretation, this observation would unphysically imply that materials that are expected to exhibit significantly different electronic properties have the same band gap.^52^ Indeed, the very sharp nature of this peak is more consistent with a vibronic origin rather than an electronic transition. We were able to identify it as the dominant vibrational line (0–0) of the S_1_ ← S_0_ transition of toluene,^73,74^ which was not entirely removed from the sample after the NCs were transferred to hexane for absorption spectroscopy (a necessary step due to the limited transparency window of toluene in the UV region). It is worth noting that all absorption spectra presented in Figure S13 are shown up to 500 nm to highlight the absence of localized surface plasmon resonance (LSPR) features in the 400–500 nm range, which have been identified in previous studies^23,34^ as characteristic signatures of Ag(0) nanoparticles in the samples.

**Figure 5:**
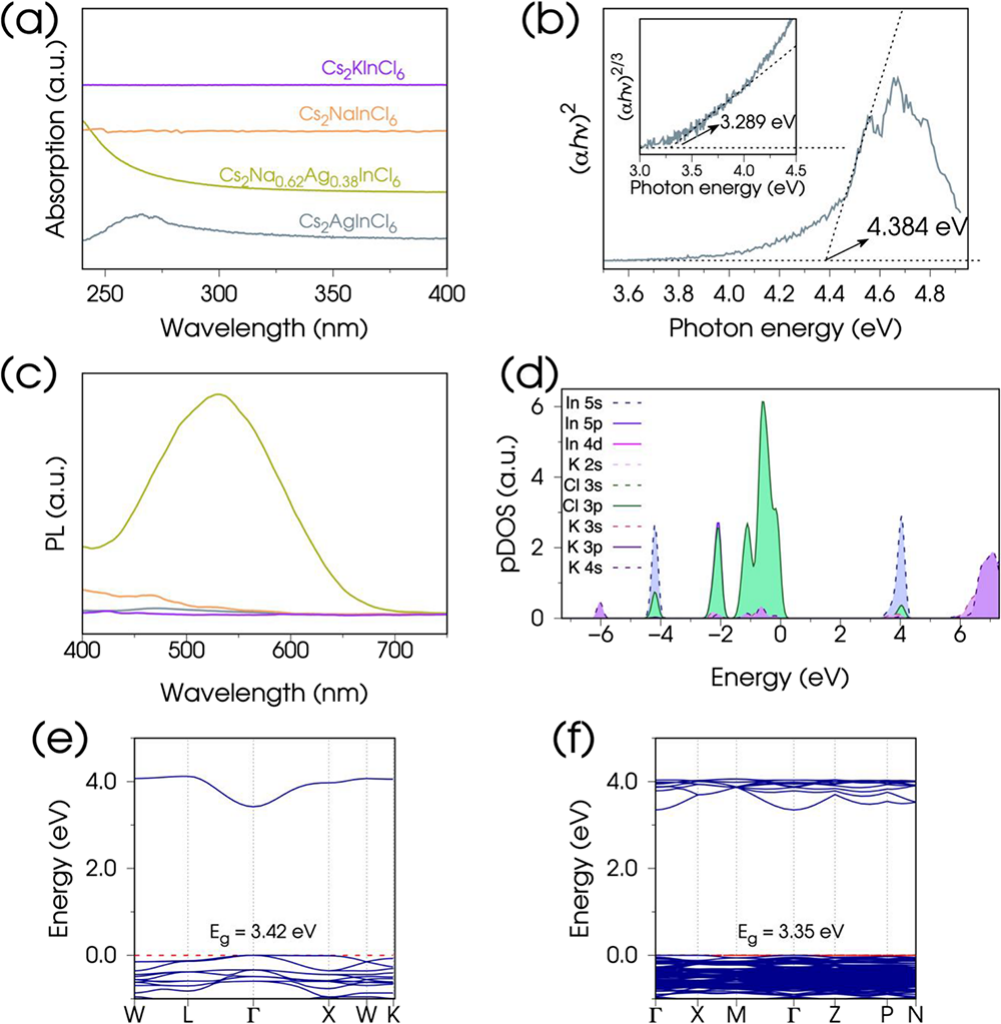
(a) UV–vis absorption spectra of selected Cs_2_B^+^InCl_6_ compositions dispersed in hexane. (b) Tauc plot of Cs_2_AgInCl_6_ NCs obtained by fitting the strong absorption tail in the 4.45–4.55 eV energy range. Inset: zoomed-in view of the lowest-energy absorption feature and the corresponding Tauc fit performed in the 3.4–4.0 eV range. (c) Photoluminescence spectra of the HDP NC compositions shown in (a), using the same color codes. (d) pDOS of Cs_2_KInCl_6_ in the tetragonal *I4/m* symmetry, with the different atomic contributions close to the band gap, aligned to the Fermi energy at 0 eV (contributions to pDOS from Cs are omitted for clarity). Band structure of Cs_2_KInCl_6_ in the cubic *Fm3̅m* (e) and tetragonal *I4/m* (f) symmetries, with the corresponding energy gaps (*E*_g_) resulting from DFT calculation and geometry optimization starting from the experimental structures (details are provided in the Methods and Supporting Information file).

In Figure 5a, we report the same absorption spectra free of toluene traces, obtained after thorough vacuum drying to ensure the complete removal of residual toluene and subsequent redispersion of the NCs in hexane (the corresponding UV–vis spectra before and after subtraction of the toluene contribution are reported in Figure S13). The absorption spectrum of Cs_2_AgInCl_6_ shows a strong peak at 266 nm followed by a broad tail extending up to ∼400 nm. Based on literature values for the bandgap of colloidal Cs_2_AgInCl_6_ NCs (ranging from 4.25 to 4.88 eV),^16,22^ we attribute the broad feature at ∼300 nm to a parity-allowed transition (Γ_4_^–^ → Γ_1_^+^) occurring between the valence band and the CBM.^35^ Figure 5b shows the fit of the experimental absorption coefficient to the Tauc equation using 2 as exponent (γ = 1/2, as detailed in the Supporting Information file), yielding an energy gap of 4.384 eV.

It is worth noting that this value, previously attributed to the optical band gap for ultrasmall colloidal NCs,^16,22^ is significantly blue-shifted compared to bulk Cs_2_AgInCl_6_, for which reported bandgap values range between 3.20 and 3.33 eV.^40,71,72^ However, in line with Lee et al.,^35^ who detected a lower-energy feature in ∼200 times concentrated colloidal suspensions, we could identify a weak absorption shoulder at ∼400 nm in our spectra. The corresponding Tauc plot (inset of Figure 5b) using an exponent of 2/3 (γ = 3/2), suggests a transition energy of 3.289 eV, consistent with the bulk optical bandgap and attributed to the (partially) forbidden transition between the VBM and the CBM. A similar analysis was performed on the mixed composition Cs_2_Na_0.62_Ag_0.38_InCl_6_ and reported in Figure S13 of the Supporting Information.

Another interesting feature observed from Figures 5a and S13 is the featureless absorption spectra of Cs_2_NaInCl_6,_ Cs_2_KInCl_6_, if compared to those of Cs_2_(Na,Ag)­InCl_6_. The chemical bond analysis provided above explains this finding, highlighting the predominantly ionic nature of the Na–Cl interaction compared to both In–Cl and Ag–Cl bonds. As showcased in Figure 3b, the pDOS of Cs_2_NaInCl_6_ is dominated by the In 5s and Cl 3p states close to the band gap, due to the minimal overlap between Na 3p and Cl 3p atomic orbitals. Similar considerations apply to the K–Cl bonds in Cs_2_KInCl_6_ (Figure 5d), as well as to all other HDPs with an alkali metal as B^+^ ion.^6^ As a result, the band edges of Cs_2_NaInCl_6_ and Cs_2_KInCl_6_ are shifted toward higher energies compared to those of Ag-containing HDPs. The computed band gap corresponding to a parity-forbidden transition^70^ is 2.92 eV for Cs_2_NaInCl_6_ (Figure S5) and 3.42 eV for Cs_2_KInCl_6_ (Figure 5e), which are respectively 1.9 and 2.4 eV higher than the value computed from Cs_2_AgInCl_6_ (0.99 eV, Figure S5). Considering the well-known underestimation of the DFT simulated band gap at the PBE level of theory^19,70^ – typically by ∼1.3 to 1.5 eV for wide-gap HDPs (as detailed in the Supporting Information) – we estimate the corrected values for the lowest-energy parity-forbidden transitions to be approximately 4.1–4.3 eV (∼302 to 288 nm) for Cs_2_NaInCl_6_ and 4.7–4.9 eV (∼263 to 253 nm) for Cs_2_KInCl_6_. Consequently, the first parity-allowed transitions, occurring at even higher energies relative to the band edges, are expected to lie near or beyond the detectable range of our experimental UV–vis setup, which explains the relatively featureless absorption spectra observed for these compositions (Figure 5a).

In Figure 5e,f, we compare the electronic band structure plots for Cs_2_KInCl_6_ computed by using the cubic *Fm3̅m* and distorted tetragonal *I4/m* symmetries, respectively. Although some differences, attributed to symmetry reduction, are found – highlighting the importance of thorough structural characterization for developing the correct theoretical framework–a direct transition with a parity-forbidden character is preserved, displaying a comparable band gap and analogous conduction and valence bands near the Γ point. Moreover, the (negligible) K–Cl contribution to the pDOS shown in Figure 5d, computed in the tetragonal model, aligns well with the one derived from the *elpasolite* structure of Cs_2_NaInCl_6_ (Figure 3b). This suggests that the peculiar coordination found for some K^+^ in Cs_2_KInCl_6_ (pentagonal bipyramid) relative to the B^+^Cl_6_ octahedra of the archetypal *elpasolite* model has minor influence on the band structure and optical properties of these materials, since the fundamentally ionic nature of the B^+^–Cl interactions is unchanged across different structural models when B^+^ is an alkali metal.

On the other hand, the mixed Cs_2_Na_0.62_Ag_0.38_InCl_6_ composition shows a very peculiar feature in its absorption spectra below 250 nm, which we highlight here as a spectral fingerprint of improved emission properties. This is demonstrated by the corresponding PL spectra in the green-yellow regions in Figure 5c, which show a maximum at 531 nm (2.33 eV) upon excitation at 260 nm, corresponding to the peak emission properties observed for these HDP NCs. A much lower PL in the green-yellow regions is also found for the Cs_2_Na_0.34_Ag_0.66_InCl_6_ mixed composition (Figure S13). The broad emission (fwhm ∼0.66 eV/147 nm) and marked Stokes (red) shift (∼1 eV/175 nm) from the band edge found for Cs_2_Na_0.62_Ag_0.38_InCl_6_ NCs (2.975 eV, Figure S13) is a characteristic feature commonly observed in the PL spectra of HDPs.^28,36,75–77^ The unique intrinsic structural modulations of HDPs, stemming from the ordered incorporation of monovalent and trivalent cations in the B-sites, in contrast to conventional ternary ABX_3_ perovskites, make these materials particularly susceptible to dynamic symmetry breaking driven by local structural distortions and transient lattice deformations, especially when combined with the wide range of possible alloying and doping strategies.^76,77^ As a result, the formation of STEs followed by radiative recombination – i.e., dark-to-bright STE conversion induced by the symmetry breaking of the excited state – has been increasingly identified as a dominant photophysical mechanism, as supported by recent ultrafast transient absorption and time-resolved photoluminescence studies.^16,17,20,21,23,27,35,36,78–85^ These insights are further corroborated by pump–probe terahertz spectroscopy,^75,83^ which together converge on the following picture: upon photoexcitation, strong electron–phonon coupling leads to rapid (∼1 ps) self-trapping of photogenerated carriers, triggering local lattice distortions and the formation of polaronic states.^75,83,86^ These structural distortions increase the ground-state energy and simultaneously decrease the excitonic energy through lattice relaxation, resulting in the broad, red-shifted emission typically observed in the PL spectra of HDPs.

Here, we report the evidence of a modification of the electronic structure in the mixed Cs_2_Na_0.62_Ag_0.38_InCl_6_ composition of HDPs NCs. This modification leads to a clear change in the absorbance features compared to the endmembers, and it is directly associated with the appearance of an enhanced PL peak in the visible region. We suggest that the Na^+^/Ag^+^ substitution creates local structural inhomogeneities (such as B^+^Cl_6_ clustering, even at the NC surface) within the otherwise ordered alternation of B^+^Cl_6_ and InCl_6_ octahedra in the equilibrium structure of Cs_2_B^+^InCl_6_. These inhomogeneities may promote dynamic symmetry breaking in the excited-state structure, a mechanism directly linked to the formation of bright STEs and radiative recombination of the excitons observed in the form of red-shifted PL (Figure 5c). One experimental piece of evidence of B^+^Cl_6_ clustering upon Na^+^/Ag^+^ alloying is provided for the Cs_2_Na_0.34_Ag_0.66_InCl_6_ stoichiometry. This composition shows deviations from the Vegard’s law for solid solutions (Figure 2b) and a broad low-angle shoulder in all reflections of the WAXTS pattern (Figure S7), indicating a (small-but detectable) deviation from the *elpasolite* average crystal structure. Moreover, we emphasize that X-ray scattering techniques *are not spatially limited* methods, as they can only detect structural modifications that affect a significant number of atoms within the (nano)­crystal structure, especially in cases where the NC sizes are not particularly small, as in this work, and if the structural changes are arranged in a nonrandom fashion. Therefore, the detection of this peculiar structural feature exclusively in the Cs_2_(Ag,Na)­InCl_6_ composition with the lowest Na^+^ fraction may be explained by a progressive randomization of the suggested NaCl_6_ clustering as the Na^+^ alloying within the AgCl_6_ sublattice increases.

## Conclusions

In summary, we have developed an alternative multistep synthetic approach for the synthesis of Cs_2_B^+^InCl_6_ colloidal NCs, characterized by high purity (nano)­crystalline phases with good stability in ambient conditions. Unlike conventional one-pot methods reported in the literature, this approach facilitates rapid and straightforward compositional engineering of the final products. It offers improved control, ensuring homogeneity of the reaction mixture during injection, while also enhancing scalability (for both small- and large-scale syntheses), reproducibility (e.g., in terms of mixture homogeneity, precise heating, and defined reaction times and temperatures), and the flexibility to explore various cationic substitutions within the 3D HDP structures. A similar approach involving the preparation of metallic stock solutions, which are mixed in the appropriate molar ratio to achieve the targeted stoichiometries, is envisioned for alloying and doping of the B^3+^ site and further exploring the thermodynamic stability of mixed halide compositions,^87^ though this is beyond the scope of the current manuscript.

Building on these favorable synthetic conditions, we have investigated the evolution of the B^+^Cl_6_ and B^3+^Cl_6_ octahedra upon (Na,Ag) alloying in the HDP B^+^ site, using detailed structural analysis of high-resolution WAXTS data sets from the corresponding colloids. Supported by DFT calculations of the charge density distributions, our findings indicate a progressive decrease in the covalent character of the B^+^–Cl bonds as we move from Ag^+^ to Na^+^, accompanied by a shrinkage of the B^3+^Cl_6_ octahedra and an expansion of the B^+^Cl_6_ ones. The insights into the nature of chemical bonding across the different HDP compositions elucidated in this work have also enabled a more detailed interpretation of the UV–vis spectral features, contributing to the clarification of previously inaccurate reports in the literature.

Moreover, the developed multistage approach has allowed us to stabilize previously underexplored HDP phases [Cs_2_(Na,K)­InCl_6_] allowing their full characterization and providing insights on K^+^ alloying within the NaCl_6_ sublattice. Atomistic details were also provided, including the distorted (partially edge-sharing) tetragonal structure of Cs_2_KInCl_6,_ together with its structural evolution with temperature variation.

Finally, the enhanced emission properties found for (Na,Ag) mixed compositions compared to the endmembers, was related to the structural evidence of NaCl_6_ clustering within the AgCl_6_ sublattice in Cs_2_AgInCl_6_ NCs. This suggests a modification of the structure of HDPs upon alloying, which may drive (dynamic) symmetry breaking in the excited state structure, thus accounting for their superior optical properties compared to their undoped counterparts. Further insights into the out-of-equilibrium structures of these materials, characterized by strong exciton–phonon coupling and STE mechanisms induced by lattice deformations, are to be specifically addressed by dedicated ultrafast studies^88^ (similar to those reported on lead-based perovskites^89–94^ ) and/or time-resolved spectroscopic experiments.

In conclusion, our findings reconcile the development of synthetic approaches for driving the design of NCs and their atomic-scale characterization using advanced structural and computational methods, paving the way for the rational development of next-generation HDP semiconductors with enhanced stability and superior optoelectronic properties.

## Methods

### Synthesis of Cs_2_B^+^InCl_6_ (with B^+^ = Ag, Na, K) NCs

For all the syntheses, a base solvent (BSv) was prepared by mixing and degassing in vacuum at 140 °C 100 mL of 1-octadecene, 6.6 mL of oleylamine, and 25 mL of oleic acid in a 250 mL two-neck round-bottom flask.

Individual stock precursor solutions were prepared for the synthesis of Cs_2_AgInCl_6_ NCs. Cesium carbonate (0.0501 g, 0.31 mmol) was dissolved in 3.1 mL of base solvent (BSv) and 1 mL of 1-octadecene (ODE) to form the Cs stock solution. The Ag stock solution was prepared by dissolving silver acetate (0.0301 g, 0.18 mmol) and triphenylphosphine (0.0407 g, 0.16 mmol) in 3.1 mL of BSv and 1 mL of ODE. Similarly, indium biacetate hydroxide (0.0384 g, 0.15 mmol) was dissolved in 3.1 mL of BSv and 1 mL of ODE to form the In stock solution. Lastly, the Cl stock solution was prepared by mixing benzoyl chloride (1 mL, 7.8 mmol) with 2 mL of BSv.

For the synthesis of Cs_2_AgInCl_6_ NCs, 1 mL each of the Cs, Ag, and In stock solutions was mixed in a vial and preheated to 70 °C with continuous stirring and degassed in vacuum for at least 10 min. Simultaneously, the Cl stock solution was also heated to 70 °C. The reaction mixture was heated to 170 °C over a period of 184 s, at which point 150 μL of the preheated Cl stock solution was rapidly injected under vigorous stirring. The reaction was quenched by cooling the vial in an ice bath after 10 s. The reaction mixture was centrifuged at 4000×*g* for 10 min. The resulting precipitate was redispersed in ODE and centrifuged again. Finally, the purified nanocrystals were centrifuged again and redispersed in toluene and stored in a closed vial under ambient conditions.

The synthesis of Cs_2_NaInCl_6_ and Cs_2_KInCl_6_ NCs followed an analogous procedure, with the only modification being the substitution of silver acetate with either sodium acetate or potassium acetate in the respective stock solutions, this time prepared without the addition of triphenylphosphine. The synthesis of Cs_2_Na_*x*_Ag_1–*x*_InCl_6_ NCs, a controlled mixture of Ag and Na stock solutions, was used to achieve a tunable composition. The molar ratios of Cs, Ag, Na, In, and Cl were set at 2:(1.17·*x*):(1–*x*):1:10.4, ensuring that the Cl precursor was used in excess and Ag was present in slight excess relative to the stoichiometric amount. In the case of Cs_2_Na_*x*_K_1–*x*_InCl_6_ NCs, only the Cl precursor was used in excess, with the final molar ratios of precursors being Cs:K:Na:In:Cl = 2:(*x*):(1–*x*):10.4.

A more complete description of the developed synthesis approach for Cs_2_B^+^InCl_6_ NCs is reported in the Supporting Information file.

The synthesized Cs_2_B^+^InCl_6_ NCs were characterized using a combination of techniques-including transmission electron microscopy (TEM), inductively coupled plasma (ICP), steady-state UV–vis absorbance and photoluminescence (PL), and wide-angle X-ray total scattering (WAXTS)-with the experimental results complemented by ab initio Density Functional Theory (DFT) calculations.

### Characterization of Cs_2_B^+^InCl_6_ NCs

Elemental concentrations were determined by an Inductively Coupled Plasma-Mass Spectrometry (ICP-MS) iCAPQ from Thermo Scientific. Prior to analysis, samples were dissolved using a 1:3 HNO_3_:HCl mixture by microwave-assisted digestion with an Ethos 1 equipment (Milestone S.r.l.). TEM measurements were performed using a JEOL 1400Plus transmission electron microscope (TEM) operating at 80 kV. Images were acquired at standard magnifications (125 and 250 K) and processed semiautomatically using a locally developed Python3 code (details are provided in the Supporting Information file). Additionally, high-resolution TEM (HRTEM) and high-angle annular dark-field (HAADF) scanning transmission electron microscopy (HAADF-STEM) were performed using TEM JEOL F200 operated at 200 kV. Elemental analysis and mapping were performed using a JEOL 100 mm^2^ silicon drift energy dispersive X-ray spectrometer (EDX). Carbon-supported copper grids, 400 mesh size, were used for sample preparation. HRTEM images were analyzed using Fiji.^95^ Steady-state UV–vis absorption spectra were recorded using a home-built setup, as detailed in the Supporting Information. The strong scattering signal in the samples, likely due to aggregates, was evident from the characteristic absorbance tail at long wavelengths. To isolate the absorption, we first modeled the scattering contribution at wavelengths longer than 450 nm- where electronic transitions are absent- using a power law function that effectively represents scattering effects.^96,97^ We extended this contribution into the absorption region and subtracted it to isolate the bare absorption (details on this procedure are given in the Supporting Information file). The PL emission spectra were recorded using a PTI Fluorescence Master System spectrofluorometer controlled by proprietary acquisition software that performs real-time corrections for the spectral responses of both the excitation lamp and the detector. Both the lamp and detector slits were set to a 4 nm aperture.

### X-ray Scattering Data Collection, Reduction and Modeling for Cs_2_B^+^InCl_6_ NCs

X-ray diffraction data for Cs_2_Na_*x*_K_1–*x*_InCl_6_ (with *x* = 0.86 and 0.78) were collected using Cu–K_α_ radiation (λ = 1.5418 Å) on a Rigaku Miniflex diffractometer equipped with a DTEX detector operating at 30 kV and 10 mA. The measured angular ranges for all data sets are characterized by a 2θ_min_ = 8° and a 2θ_max_ = 80°, with a 2θ-step of 0.1°.

For all the other HDP compositions, wide-angle X-ray total scattering (WAXTS) synchrotron data were collected on both colloidal and dry samples at the high-resolution powder diffraction beamline (ID22) of the European Synchrotron Radiation Facility (ESRF, Grenoble, France)^98^ using a 35 keV beam in the 0.01°–82° 2θ range. Data were collected with a 13-channel Si 111 multianalyzer stage coupled with a single photon-counting Dectris Eiger2 × 2M-W CdTe pixel detector. High-temperature measurements on Cs_2_KInCl_6_ were performed through a temperature controlled N_2_ stream fluxing over the capillary.

Detailed procedures on the thorough data reduction-including corrections for absorption, capillary, and air scattering, as well as the structural analysis carried out using the Rietveld method and TOPAS software^99^ are provided in the Supporting Information file.

### Density Functional Theory (DFT) Calculations

All Density Functional Theory (DFT) calculations including full geometry optimization were performed using the Quantum ESPRESSO software package,^100–102^ employing the projector augmented wave (PAW) method.^103^ The Perdew–Burke–Ernzerhof (PBE) exchange-correlation functional within the generalized gradient approximation (GGA) was implemented.^59^ The Brillouin zone was sampled with a Γ-centered Monkhorst–Pack grid corresponding to a k-point spacing of approximately 0.15 Å^–1^. More details are provided in the Supporting Information file.

## Supplementary Material





## Footnotes

* [*a*_400K_ – *a*_RT_]/ *a*_RT_ × 100, where *a*_400K_ = 10.93009(1) Å and *a*_RT_ = 10.82097(3) Å are the cubic and equivalent cubic lattice parameters at 400 K and RT, respectively.

## Notes

Crystallographic Information Files for Cs_2_(Na,Ag)­InCl_6_ structures refined from room temperature high-resolution synchrotron WAXTS data: CSD deposition nr. 2433761, 2433762, 2433765, Cs_2_AgInCl_6_ and Cs_2_NaInCl_6._ Crystallographic Information Files for and Cs_2_KInCl_6_ structures refined from both room temperature and 400 K high-resolution synchrotron WAXTS data: CSD deposition nr. 2433763 and 2433764. Supporting Information file detailing the optimization of the synthetic strategy, the structural characterization of Cs_2_B^+^InCl_6_ colloidal NCs (WAXTS data collection, reduction, and Rietveld modeling), density functional theory calculations, optical characterization, and transmission electron microscopy characterization details. Tables S1–S5 report the numerical details, and figures S1–S12 the graphical outcome related to the supplementary text (PDF)
Crystallographic information files for Cs_2_(Na,Ag)­InCl_6_ structures refined from room-temperature high-resolution synchrotron WAXTS data: CSD deposition nr. 2433761, 2433762, 2433765, Cs_2_AgInCl_6_ and Cs_2_NaInCl_6_; and crystallographic information files for Cs_2_KInCl_6_ structures refined from both room-temperature and 400 K high-resolution synchrotron WAXTS data: CSD deposition nr. 2433763 and 2433764 (ZIP)
