# Supplementary material for: Atomistic Insights into Halide Double Perovskite Nanocrystals obtained by Multistep Synthesis and Efficient Compositional Engineering
Source: ACS Nano. 2025 Aug 11;19(33):30151–64. doi: 10.1021/acsnano.5c06497 (PMC12392739; doi:10.1021/acsnano.5c06497)
Supplement: Supplementary file 1 [file nn5c06497_si_001.pdf]

Supporting Information for

Atomistic Insights into Halide Double Perovskite Nanocrystals obtained by Multi-step Synthesis and Efficient Compositional Engineering

Nicola Dengo,[1,2,3] *David F. Macias-Pinilla,*[4] *Pietro Anzini,* [1,2,5] *Mara Colombo,* [1,2] *Simone Virga,*[4] *Andrea Brambilla,*[6] *Piero Antonio Zecca,*[6] *Damiano Monticelli,*[1] *Francesco Giannici,**[4] *and Federica Bertolotti**[1,2]

[1] Department of Science and High Technology, University of Insubria, via Valleggio 11, Como 22100, Italy.

[2] *Total Scattering Laboratory* (To.Sca.Lab), Department of Science and High Technology, University of Insubria, via Valleggio 11, Como 22100, Italy.

[3] Department of Chemical Sciences, University of Padova, via Marzolo 1, Padova 35131, Italy.

[4] Department of Physics and Chemistry "Emilio Segrè", University of Palermo, viale delle Scienze, Ed.17, Palermo 90128, Italy.

[5] CLIP-Como Lake Institute of Photonics, via Valleggio 11, 22100 Como, Italy.

[6] Department of Medicine and Technological Innovation, University of Insubria, via Guicciardini 9, Varese 21100, Italy.

1. *Synthetic strategy optimization* ... 3
Materials ... 4
Preparation of the base solvent ... 4
Preparation of Na, K, In, and Cs stock precursor solutions ... 4
Preparation of Ag stock precursor solutions ... 4
Preparation of Cl stock precursor solutions ... 5
Synthesis of $Cs_2AgInCl_6$ NCs ... 5
Synthesis and purification of $Cs_2NaInCl_6$ NCs ... 5
Synthesis and purification of Cs_2KInCl_6 NCs ... 5
Synthesis and purification of $Cs_2Ag_xNa_{1-x}InCl_6$ NCs ... 6
Synthesis and purification of $Cs_2Na_xK_{1-x}InCl_6$ NCs ... 6
2. *Structural characterization of $Cs_2B^+InCl_6$ colloidal NCs* ... 7
Synchrotron wide angle X-ray Total Scattering data collection and reduction ... 7
Synchrotron WAXTS data modeling ... 8
3. *Density Functional Theory calculations* ... 8
Theoretical methods ... 8
Decomposition Enthalpy ... 8
Band Structure calculations ... 9
Electron Density and Empirical Atomic Radii ... 9
4. *Optical characterization of $Cs_2B^+InCl_6$ colloidal NCs* ... 10
5. *Transmission Electron Microscopy (TEM)* ... 11
6. *Supplementary Tables* ... 12
7. *Supplementary Figures* ... 15

1. Synthetic strategy optimization

In this work, we developed a synthetic strategy to prepare halide double perovskite (HDP) nanocrystals (NCs) via solution-based synthesis. Conventional hot-injection procedures typically employ one-pot strategies in which precursors - often introduced as salts along with the reaction solvent, ligands, or liquid precursors - are dissolved by heating, stirring, and degassing the reaction mixture at a temperature lower than the injection temperature. Alternatively, the reaction mixture may be maintained at the injection temperature for an extended period to promote dissolution. In contrast, the strategy presented herein separates the dissolution and reaction stages. Initially, precursor solutions are prepared, each typically containing only one of the elements required for the target NC composition. These precursor solutions are subsequently mixed in appropriate proportions to achieve the desired concentrations and molar ratios.

The advantages of this approach include:

1. Improved reaction control. Ensures that the reaction mixture is homogeneous at the time of injection. This is critical, particularly in systems where side reactions can obscure the detection of residual undissolved precursors, as in the case of the reduction of Ag(I) precursors to Ag(0).
2. Expanded reaction conditions. Allows for a broader range of reaction parameters (e.g., reaction temperatures lower than the dissolution temperature).
3. Improved scalability. Facilitates both small- and large-scale synthesis. In particular, small-scale synthesis is no longer constrained by the minimum measurable quantity of solid-state precursors in a typical laboratory.
4. Increased applicability to flow chemistry. Adapts well to flow chemistry techniques, including microfluidics.
5. Faster synthesis. Reduces the number of time-consuming steps (e.g., precise weighing and dissolving of solid precursors), as simple mixing of solutions replaces these procedures.
6. Simpler development of self-driving or automated laboratories. Simplifies the implementation of self-driving laboratory platforms for HDP systems by using stock solutions instead of solid precursors.[1–4]
7. Potentially improved reproducibility. Offers enhanced control over reaction conditions (e.g., mixture homogeneity, precise heating, and defined reaction times and temperatures).

In this work, we piloted the development of this approach by targeting well-known compositions with the general formula $Cs_2(Ag,Na)InCl_6$ and further extending our investigation to the $Cs_2(Na,K)InCl_6$ system, which has only been superficially explored. The synthetic system employed oleic acid and oleylamine, which are widely used in the synthesis of a wide plethora of NCs. The chosen solvent was 1-octadecene, the most commonly employed solvent for the synthesis of HDP NCs via hot-injection approaches and generally less expensive than alternatives such as dioctyl ether. The reaction protocol is subdivided into the following steps:

1. Preparation of the base solvent.
2. Preparation of stock precursor solutions.
3. Preparation of the reaction mixture by mixing stock precursor solutions.
4. Synthesis of the HDP NCs via hot injection.

5. Purification through consecutive cycles of centrifugation and redispersion.

Materials

1-octadecene (ODE, Sigma-Aldrich, 90%), oleic acid (OA, Sigma-Aldrich, 90%), oleylamine (OAm, Sigma-Aldrich, ≥ 90%), toluene (Carlo Erba, anhydrous), cesium carbonate (Cs_2CO_3, Sigma-Aldrich, 99.9% trace metal basis), sodium acetate [Na(OAc), Sigma-Aldrich, anhydrous ≥ 99.0%], silver acetate [Ag(OAc), Sigma-Aldrich, 99%], potassium acetate [K(OAc), Sigma-Aldrich, ≥ 99%], triphenylphosphine (PPh3, Roth, for synthesis ≥ 99.5%), indium biacetate hydroxide [$In(OAc)_2OH$, Sigma-Aldrich. This precursor was sold by the supplier as indium triacetate. The composition here reported was determined analytically], benzoyl chloride (BzCl, Sigma-Aldrich, for synthesis)

Preparation of the base solvent

The stock solution of the base solvent (BSv) was prepared by mixing 100 mL of 1-octadecene (ODE), 6.6 mL of oleylamine (OAm; 20 mmol), and 25 mL of oleic acid (OA; 79 mmol) in a 250 mL two-neck round-bottom flask. The target molar ratio of OA to OAm was 3.9:1. The mixture was stirred, heated in an oil bath at 140 °C, and degassed under vacuum (~75 Torr) for 20 minutes, after which the solution turned a light yellow. This same BSv stock solution was used for all subsequent preparations.

Preparation of Na, K, In, and Cs stock precursor solutions

Stock solutions of the target elements were prepared by individually dissolving the precursor salts in an appropriate volume of BSv. The solutions were prepared in vials sized relative to the target volume. For example, to prepare a stock solution with a target volume of approximately 4.1 mL, 10 mL vials were used. The following procedures were employed:

- Na Stock Solution: 0.0126 g (0.15 mmol) of Na(OAc) was dissolved in 3.1 mL of BSv and 1 mL of ODE.
- K Stock Solution: 0.0151 g (0.15 mmol) of K(OAc) was dissolved in 3.1 mL of BSv and 1 mL of ODE.
- In Stock Solution: 0.0384 g (0.15 mmol) of $In(OAc)_2OH$ was dissolved in 3.1 mL of BSv and 1 mL of ODE.
- Cs Stock Solution: 0.0501 g (0.31 mmol) of Cs_2CO_3 was dissolved in 3.1 mL of BSv and 1 mL of ODE.

Dissolution was achieved by stirring the mixtures while heating them in an oil bath at 140 °C. Vacuum was not applied, as its use was found to promote gel formation upon cooling the solutions to room temperature. In the cases of the Cs and In stock solutions, heating resulted in a light brown-orange coloration. The stock solutions were stored in air and room temperature and remained stable for several days without apparent changes.

Preparation of Ag stock precursor solutions

The Ag stock solution was prepared by dissolving the precursor salts Ag(OAc) in an appropriate volume of BSv. For example, to prepare a stock solution with a target volume of approximately 4 mL, 0.0301 g (0.18 mmol) of Ag(OAc) and 0.0407 g (0.16 mmol) of PPh_3 were added to a 10 mL vial, followed by 3.1 mL of BSv and 1 mL of ODE. The solids were dissolved by stirring at room temperature. If full dissolution was not achieved after several hours, the mixture was gently heated in

a 70 °C oil bath until complete dissolution was obtained. The stock solution was then stored in air at room temperature and remained stable for several days without any apparent changes.

Preparation of Cl stock precursor solutions

The Cl stock solution was prepared by mixing the Cl precursor and BSv. For example, to prepare a 3 mL stock solution, 1 mL (7.8 mmol) of BzCl was mixed at room temperature with 2 mL of BSv in a 10 mL vial. The resulting solution turned a light yellow-pinkish color.

Synthesis of $Cs_2AgInCl_6$ NCs

In a typical synthesis, 1 mL of the Cs stock solution, 1 mL of the Ag stock solution, and 1 mL of the In stock solution were combined in a 10 mL vial. The molar ratios of the precursors were Cs:Ag:In:Cl = 2:1.17:1:10.4, thus the Cl precursor was used in excess, and Ag was used in slight molar excess. The resulting solution was preheated and stirred in an oil bath at 70 °C and degassed in vacuum (~75 Torr) for at least 10 minutes. Simultaneously, the Cl stock solution was also heated to 70 °C. The reaction mixture was then raised to 170 °C over a heating period of 184 seconds, at which point 150 μL of the preheated Cl stock solution was swiftly injected into the vial under vigorous stirring. After 10 seconds, during which the mixture turned brownish-yellow and became opaque, the reaction was quenched by immersing the vial in an ice water bath.

For purification, the reaction product was transferred to a 15 mL centrifuge tube and centrifuged for 10 minutes at 4000 × g (RCF). The supernatant was discarded, and the precipitate was redispersed in ODE using a volume equal to that of the reaction mixture employed for purification. The suspension was centrifuged again for 10 minutes at 4000 × g, the supernatant was discarded, and the precipitate was finally redispersed in toluene (using the same volume as the previously used ODE). The resulting suspension was stored in a closed vial under ambient conditions.

Synthesis and purification of $Cs_2NaInCl_6$ NCs

In a typical synthesis, 1 mL of the Cs stock solution, 1 mL of the Na stock solution, and 1 mL of the In stock solution were combined in a 10 mL vial. The molar ratios of the precursors were Cs:Na:In:Cl = 2:1:1:10.4, thus the Cl precursor was used in excess. The resulting solution was preheated and stirred in an oil bath at 70 °C and degassed in vacuum (~75 Torr) for at least 10 minutes. Simultaneously, the Cl stock solution was also heated to 70 °C. The reaction mixture was then raised to 170 °C over a heating period of 184 seconds, at which point 150 μL of the preheated Cl stock solution was swiftly injected into the vial under vigorous stirring. After 10 seconds, during which the mixture turned white and became opaque, the reaction was quenched by immersing the vial in an ice water bath.

For purification, the reaction product was transferred to a 15 mL centrifuge tube and centrifuged for 10 minutes at 4000 × g (RCF). The supernatant was discarded, and the precipitate was redispersed in ODE using a volume equal to that of the reaction mixture employed for purification. The suspension was centrifuged again for 10 minutes at 4000 × g, the supernatant was discarded, and the precipitate was finally redispersed in toluene (using the same volume as the previously used ODE). The resulting suspension was stored in a closed vial under ambient conditions.

Synthesis and purification of Cs_2KInCl_6 NCs

In a typical synthesis, 1 mL of the Cs stock solution, 1 mL of the K stock solution, and 1 mL of the In stock solution were combined in a 10 mL vial. The molar ratios of the precursors were Cs:K:In:Cl

= 2:1:1:10.4, thus the Cl precursor was used in excess. The resulting solution was preheated and stirred in an oil bath at 70 °C. Simultaneously, the Cl stock solution was also heated to 70 °C and degassed in vacuum (~75 Torr) for at least 10 minutes. The reaction mixture was then raised to 170 °C over a heating period of 184 seconds, at which point 150 µL of the preheated Cl stock solution was swiftly injected into the vial under vigorous stirring. After 10 seconds, during which the mixture turned white and became opaque, the reaction was quenched by immersing the vial in an ice water bath.

For purification, the reaction product was transferred to a 15 mL centrifuge tube and centrifuged for 10 minutes at 4000 × g (RCF). The supernatant was discarded, and the precipitate was redispersed in ODE using a volume equal to that of the reaction mixture employed for purification. The suspension was centrifuged again for 10 minutes at 4000 × g, the supernatant was discarded, and the precipitate was finally redispersed in toluene (using the same volume as the previously used ODE). The resulting suspension was stored in a closed vial under ambient conditions.

Synthesis and purification of $Cs_2Ag_xNa_{1-x}InCl_6$ NCs

In a typical synthesis, 1 mL of the Cs stock solution, x mL of the Ag stock solution, (1-x) mL of the Na stock solution, and 1 mL of the In stock solution were combined in a 10 mL vial (with $0 < x < 1$, depending on the target stoichiometry). The molar ratios of the precursors were are Cs:Ag:Na:In:Cl = 2:(1.17·x):(1-x):10.4, thus the Cl precursor was used in excess, and Ag was used in slight molar excess relative to the stoichiometric amount. The resulting solution was preheated and stirred in an oil bath at 70 °C and degassed in vacuum (~75 Torr) for at least 10 minutes. Simultaneously, the Cl stock solution was also heated to 70 °C. The reaction mixture was then raised to 170 °C over a heating period of 184 seconds, at which point 150 µL of the preheated Cl stock solution was swiftly injected into the vial under vigorous stirring. After 10 seconds the reaction was quenched by immersing the vial in an ice water bath.

For purification, the reaction product was transferred to a 15 mL centrifuge tube and centrifuged for 10 minutes at 4000 × g (RCF). The supernatant was discarded, and the precipitate was redispersed in ODE using a volume equal to that of the reaction mixture employed for purification. The suspension was centrifuged again for 10 minutes at 4000 × g, the supernatant was discarded, and the precipitate was finally redispersed in toluene (using the same volume as the previously used ODE). The resulting suspension was stored in a closed vial under ambient conditions.

Synthesis and purification of $Cs_2Na_xK_{1-x}InCl_6$ NCs

In a typical synthesis, 1 mL of the Cs stock solution, x mL of the K stock solution, (1- x) mL of the Na stock solution, and 1 mL of the In stock solution were combined in a 10 mL vial (with $0 < x < 1$, depending on the target stoichiometry). The molar ratios of the precursors are Cs:K:Na:In:Cl = 2:(x):(1-x):10.4, thus the Cl precursor was used in excess. The resulting solution was preheated and stirred in an oil bath at 70 °C and degassed in vacuum (~75 Torr) for at least 10 minutes. Simultaneously, the Cl stock solution was also heated to 70 °C. The reaction mixture was then raised to 170 °C over a heating period of 184 seconds, at which point 150 µL of the preheated Cl stock solution was swiftly injected into the vial under vigorous stirring. After 10 seconds the reaction was quenched by immersing the vial in an ice water bath.

For purification, the reaction product was transferred to a 15 mL centrifuge tube and centrifuged for 10 minutes at 4000 × g (RCF). The supernatant was discarded, and the precipitate was redispersed in ODE using a volume equal to that of the reaction mixture employed for purification. The suspension

was centrifuged again for 10 minutes at 4000 × g, the supernatant was discarded, and the precipitate was finally redispersed in toluene (using the same volume as the previously used ODE). The resulting suspension was stored in a closed vial under ambient conditions.

Effect of water contamination

The synthesis, workup, and storage of the samples were carried out using standard closed-vessel techniques (i.e., common vacuum glassware and vials sealed with PTFE/silicone septa), with substance transfers performed without special precautions. This suggests that the synthesis is tolerant to moisture exposure from ambient air. However, it was observed that the presence of excess residual water in solvents or precursors led to increased formation of hydrated side products, such as $Cs_2InCl_5(H_2O)$. Therefore, it is recommended to store solvents and precursors under anhydrous conditions (e.g., storing salts in desiccators).

2. Structural characterization of $Cs_2B^+InCl_6$ colloidal NCs

Synchrotron wide angle X-ray Total Scattering data collection and reduction

Halide double perovskite (HDPs) colloidal nanocrystals (NCs) in toluene, with composition Cs_2BInCl_6 (with B = Ag^+,Na^+,K^+) were loaded into borosilicate glass capillaries with certified composition (Hilgenberg GmbH Ø = 0.5/0.8 mm). Wide Angle X-ray Total Scattering (WAXTS) measurements were performed at the high-resolution powder diffraction beamline (ID22) of the European Synchrotron Radiation Facility (ESRF, Grenoble, France).[5]

A beam energy of 35 KeV was set, and the operational wavelengths (0.354641Å) were accurately determined using a NIST powder standard. Data were collected in the 0.01°-82° 2θ range with a 13-channel Si 111 multi-analyzer stage coupled with a single photon counting Dectris Eiger2 X 2M-W CdTe pixel detector.

Air background, empty glass capillary, and pure toluene TS patterns were independently collected under the same experimental conditions.

For data reduction, angle-dependent intensity corrections were applied to the raw data to account for sample attenuation due to absorption effects and for properly removing extra-sample scattering; sample absorption curves were determined by measuring both the transmitted beam from the filled capillaries and the direct beam, while for the empty capillaries the X-ray attenuation coefficient was computed using their nominal compositions.[6,7] Air and (absorption-corrected) capillary scattering contributions were subtracted from the sample and pure solvent signals.

A temperature-controlled N_2 stream fluxing on the capillaries was used for the 295-400 K data collection performed for Cs_2KInCl_6 colloid dried inside the glass capillary.

The experimental data for $Cs_2Na_xK_{1-x}InCl_6$ [with x(Na)=0.86 and 0.78] were collected using Cu-Kα radiation (λ=1.5418 Å) on a Rigaku Miniflex diffractometer equipped with a DTEX detector operating at 30 kV and 10 mA. A droplet of each colloidal sample was deposited on the surface of a silicon monocrystal zero-background plate with the aid of a micropipette and dried in air within minutes. The measured angular ranges for all datasets are characterized by a $2\theta_{min}$= 8° and a $2\theta_{max}$ = 80°, with a 2θ-step of 0.1°.

Synchrotron WAXTS data modeling

Rietveld refinement was performed on room temperature (RT) and temperature-dependent high-resolution X-ray TS data collected on HDPs colloids and dry NCs. The refinements were carried out in the 0.7 $Å^{-1} \leq Q \leq 10.7$ $Å^{-1}$ range using the TOPAS software.[8] All the refined parameters are synoptically collected in Table S3. Figures S5, S6, S9, and S10 present the corresponding best fit for the $Cs_2(Na,Ag)InCl_6$ and $Cs_2(Na,K)InCl_6$ colloidal and dry WAXTS data, modeled using the archetypal cubic *Fm-3m* elpasolite structure-except for Cs_2KInCl_6 which was refined using a newly identified distorted tetragonal *I4/m* structure, starting from the isostructural $CsInCl_3$[9], as detailed in the main text. A comparison between the fits for the different tetragonal models (*I4/m* distorted, and "regular", according to the group-subgroup relationship, *P4/mnc* and *I4/m* structures)[10] for Cs_2KInCl_6, based on RT data from the dried sample, is shown in Figure S11. Additionally, the Rietveld refinement of the 400 K dataset confirms the structural evolution to a cubic *Fm*-3*m* phase at T > 300K (Figure S11).

For all the samples, the size-induced peaks broadening was modeled using a convolution of a Gaussian and Lorentzian functions. An additional Lorentzian function (full-width at half maximum β_L)[11] was used to account for an angle-dependent peak broadening ($\beta_L = 4\,\varepsilon \tan\theta$, where $\varepsilon = \frac{\Delta d}{d}$ is the microstrain parameter), particularly relevant for the Ag- rich HDPs, as reported in Table S3. Furthermore, a related, angle-dependent peak shift correction was applied to these samples using a phenomenological function $\Delta 2\theta = \frac{p}{cos\theta}$, where p is a refined parameter. The resulting values were -0.018(2), -0.0061(2) and -0.0095(2) for $Cs_2AgInCl_6$, $Cs_2Na_{0.34}Ag_{0.66}InCl_6$ and $Cs_2Na_{0.62}Ag_{0.38}InCl_6$, respectively. Further investigations, including local structural techniques such as X-ray absorption spectroscopies, are ongoing to clarify the underlying origin of this phenomenon.

3. Density Functional Theory calculations

Theoretical methods

Scalar relativistic PAW pseudopotentials with the PBE exchange-correlation functional were chosen from PSLibrary.[12] The general cutoff for the wavefunction is set to 100 Ry, and for the charge density cutoff, it is set to 400 Ry. The convergence of the cutoffs was tested for each structure. The SCF convergence threshold was defined as 1.0×10^{-6} Ry, and the optimized structures are relaxed until the forces on individual atoms are below 0.001 Ry/a_0.

Decomposition Enthalpy

The PBE functional has demonstrated consistency compared to more computationally expensive functionals and is commonly used in thermodynamic stability calculations.[13,14] We employed the PBE functional to calculate the total energies of the HDP materials and their decomposition enthalpy (ΔH), which is defined as the energy difference between the total energy (E_T) of the pristine material and the sum of the total energies of its most likely decomposition products:

$$\Delta H = E_T[products] - E_T[HDP]$$

Negative ΔH values indicate a tendency for the material to decompose into its products, while positive ΔH values suggest a low likelihood of decomposition. The greater the absolute values of ΔH, the higher the probability of decomposition (for negative values) or stability (for positive values). Since

HDP materials are composed of multiple cations, it is necessary to calculate the decomposition enthalpy into ternary and binary compounds to evaluate the material's stability.

We conducted a comparative study of thermodynamic stability based on the decomposition enthalpy for $Cs_2(Na^+, Ag^+, K^+)In(Cl, Br)_6$, as shown in Figure S4. In Figure S4, it can be observed that perovskites containing Cl consistently exhibit positive decomposition enthalpies toward binary or ternary compounds, indicating considerable stability. In contrast, perovskites with Br show lower decomposition enthalpy values, with some cases even presenting negative values, suggesting a higher propensity for decomposition. Therefore, from a theoretical perspective, Cl-based perovskites are more stable than their Br-based counterparts.

Band Structure calculations

The electronic structure and projected density of states were calculated using the PBE functional, which is well known to underestimate band gap values. However, it is reliable for describing electronic states and the morphology of electronic bands.[13–15] Dipole transition matrix elements, which are directly associated with the transition probability, are calculated at each k-point between the top of the valence band and the bottom of the conduction band along the path in the Brillouin zone.

The electronic bands and dipole transition elements of $Cs_2(Na, Ag, K)InCl_6$ are showed in Figure S5 and S8.

In Figures S4 and S8, it can be observed that these materials exhibit a direct band gap with forbidden transitions at the Γ point.

By comparing the simulated fundamental bandgaps at the PBE level (Figure S5) with the (average) optical bandgaps reported in the literature, these are all underestimated by about 1-2 eV, the agreement being better for more insulating compounds.

	Optical bandgap (eV)	PBE Bandgap (eV)	Underestimation (eV)
$Cs_2AgInCl_6$	3.289✧	0.99	2.30
$Cs_2NaInCl_6$	3.70-4.45[16–18]	2.92	0.78-1.53
Cs_2KInCl_6	4.62[19]	3.42	1.20

✧ *This work* (Figure 5b of the main text)

In the case of the mixed Na- and Ag-based compositions (Figure S5), there is an increase in dipole transition elements in the L-Γ and Γ-X paths, which has been experimentally observed in relation to an increase in photoluminescence quantum yield.[20,21] Moreover, an increase in the transition moments is also observed for the Na and K composition in the L-Γ path (Figure S9).

Electron Density and Empirical Atomic Radii

We calculated the electron density of the perovskites to estimate the interatomic distances between cations. In the electron density maps, the distance at which the charge density reaches a minimum between atoms is assumed as the ionic radius. In this way, by analyzing the charge distribution, one indirectly obtains the covalent or ionic character of the atomic bonds in the perovskite lattice.

4. Optical characterization of $Cs_2B^+InCl_6$ colloidal NCs

The UV-Vis absorption spectra were recorded by using a custom setup sketched in the figure below.

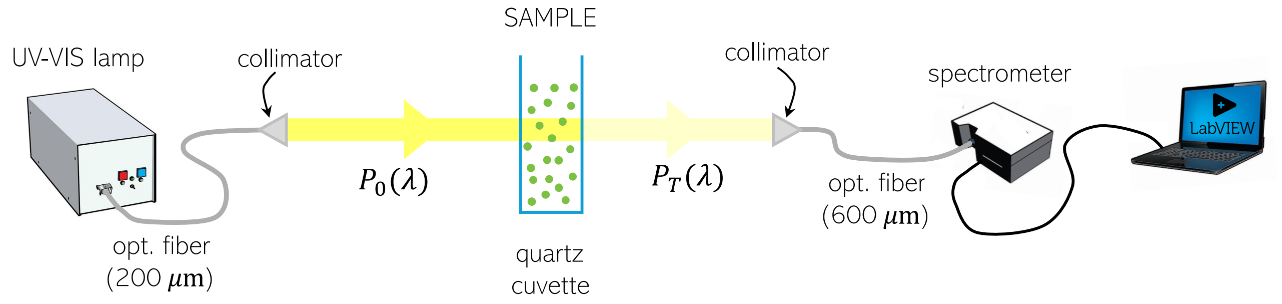

The incident, white light beam, produced by a lamp (Ocean Insights, mod DH2000-BAL) is directed perpendicularly toward the quartz sample cuvette (Hellma 110-10-40, optical path length $L = 1$ cm) using an optical fiber (Ocean Insight QP200-2-SR-BX) and a collimator (Ocean Insight 74-ACR). The transmitted beam is then collected by a second collimator and to a fiber (Ocean Insight QP600-2-SR-BX) and is transmitted to a spectrophotometer (Ocean Optics HR2000-GC-UV-NIR) operating in the $200 - 1100$ nm range. All the samples were diluted in hexane (n-Hexane for spectroscopy Uvasol® CAS 110-54-3). The spectrometer was interfaced by a custom acquisition software written in LabVIEW. For each measurement, the signal of the pure solvent in the same cuvette has also been recorded for the subtraction of the contribution of the solvent.

The absorbance measurement of a colloidal dispersion is based on the Lambert-Beer law that relates the power $P_0(\lambda)$ incident on the sample to the transmitted power $P_T(\lambda)$ by

$$P_T(\lambda) = P_0(\lambda)\, e^{-\mu_e(\lambda)L}$$

where λ is the vacuum wavelength of the incident radiation, L the sample optical path, and

$$\mu_e(\lambda) = \mu_a(\lambda) + \mu_s(\lambda)$$

is the λ-dependent *extinction* coefficient, measured in inverse centimeters, given by the sum of the *absorption* (μ_a) and *scattering* (μ_s) coefficients. Equation (1) is often reported as

$$P_T(\lambda) = P_0(\lambda)\, 10^{-ABS(\lambda)}$$

where $ABS(\lambda) = \frac{\mu_e(\lambda)L}{\ln 10}$ is the sample *absorbance*. Although it is common to associate $ABS(\lambda)$ to the bare *absorption* (related to $\mu_a(\lambda)$), the quantity that is measured also includes the contribution due to the scattering.

In the dilute limit and in the absence of large particles or aggregates, absorption essentially corresponds to absorbance, as the scattering contribution becomes negligible. However, after dilution, the samples exhibited a significant scattering signal, likely caused by the presence of aggregates. This was particularly noticeable from the characteristic tail in the absorbance at long wavelengths. A similar situation was observed for the other samples.

It is expected that, in the presence of submicrometric objects, the contribution of scattering to absorbance takes the functional form of a power law function:[22,23]

$$\mu_s(\lambda) \sim \lambda^{-\alpha}$$

where α is an exponent that, in the case of fractal objects equals $D - 4$, where D is the fractal dimension of the aggregate.

To extract the absorbance contribution due to electronic transitions, i.e., the absorption, we fitted the absorbance at long wavelengths (greater than 450 nm), where no electronic transitions occur, using

a power law function that accurately accounts for scattering. We then extrapolated the fitted values to the region where absorption is present and finally subtracted this contribution from the overall absorbance data.
In this plot, the raw data, *i.e.* the absorbance, is shown as a green line, while the scattering contribution is represented by a yellow line, which corresponds to a power law fit. The absorption, obtained by subtracting the scattering contribution from the absorbance, is depicted as a red line.

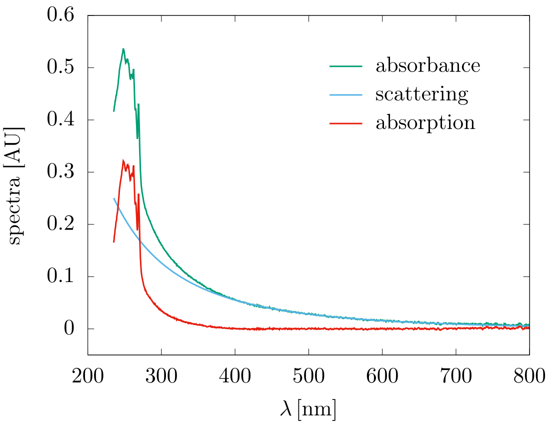

UV-Vis absorption spectra allow for the direct estimation of the Tauc exponent by fitting the experimental absorption coefficient to the Tauc equation:[24]

$$[\mu_a(\lambda)h\nu]^{1/\gamma} = A(h\nu - E_g)$$

where γ is the Tauc exponent, allowed, transitions, and E_g the optical band gap. For direct, allowed, transitions with $\gamma = 1/2$, an example of the procedure is illustrated in the figure below. In the image, the rescaled data is represented in green, the fitted data in light blue, and the solid line corresponds to the Tauc fitting.

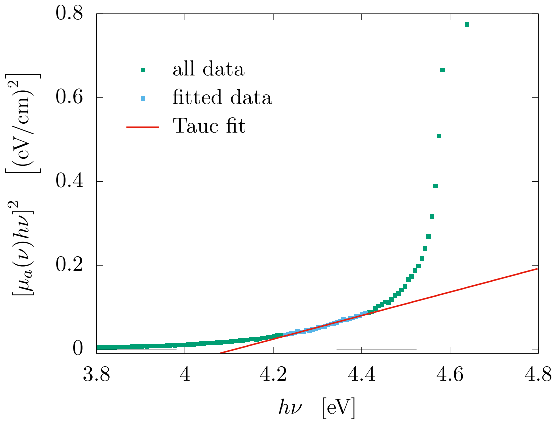

5. Transmission Electron Microscopy (TEM)

Samples were prepared by dipping carbon film-coated 300 mesh per inch gold grids for TEM in dilute suspensions of NCs in toluene. The solvent in the suspension was removed by allowing it to evaporate from the grids exposed to air. Measurements were carried out on a JEOL 1400Plus TEM operating at 80 kV using standard magnifications (125K and 250K).
TEM images were processed semi-automatically with a locally developed Python3 code. For each image, the grayscale input was first subjected to Gaussian filtering with an appropriate kernel size to reduce noise. The

filtered image was then binarized using Otsu's thresholding method, optionally modified by an offset to better capture contrast variations. A morphological opening operation, also with an appropriate kernel size, was applied to remove residual noise and small artifacts. NCs were identified algorithmically on the binarized images by fitting minimum-area bounding rectangles to each candidate region. The resulting set of rectangles was filtered based on defined thresholds for minimum and maximum area, as well as aspect ratio and tolerance. The selected rectangles were superimposed on the original image for visual validation. The area of the rectangles was then used to calculate the edge length of squares having the same area (equivalent squares). Finally, the side length distribution of these equivalent squares was fitted to a Gaussian distribution to determine the average equivalent side length and the standard deviation for each sample. The resulting average sizes and standard deviations are reported in Figure S1.

6. Supplementary Tables

Table S1. Results of the determinations of Na, K, Cs , Ag and In by Inductively Coupled Plasma - Mass Spectrometry (ICP-MS). Data are reported as molar ratios with respect to In.

	Cs	Ag	Na	In
$Cs_2NaInCl_6$	1.99	/	0.66	1.00
$Cs_2Na_{0.82}Ag_{0.18}InCl_6$	2.01	0.18	0.81	1.00
$Cs_2Na_{0.62}Ag_{0.38}InCl_6$	1.98	0.43	0.58	1.00
$Cs_2Na_{0.34}Ag_{0.66}InCl_6$	1.94	0.65	0.59	1.00
$Cs_2AgInCl_6$	1.88	1.47	/	1.00
	Cs	K	Na	In
$Cs_2Na_{0.86}K_{0.08}InCl_6$	2.15	0.08	0.86	1.00
$Cs_2Na_{0.78}K_{0.26}InCl_6$	2.25	0.23	0.78	1.00
Cs_2KInCl_6	1.98	0.87	/	1.00

Table S2. DFT-computed total energy for all compounds.

	**Total Energy (eV/atom)**		**Total Energy (eV/atom)**
$CsAgCl_2$	-3163.6515	$CsAgBr_2$	-5691.1400
Cs_2AgCl_3	-3232.4564	Cs_2AgBr_3	-5759.9434
$Cs_3In_2Cl_9$	-2705.9999	$Cs_3In_2Br_9$	-5955.5727
Na_3InCl_6	-1653.9861	Na_3InBr_6	-4686.7268
K_3InCl_6	-1669.4503	K_3InBr_6	-4702.2869
AgCl	-2957.2100	AgBr	-5484.7414
CsCl	-3369.9750	CsBr	-5897.4431
$InCl_3$	-2207.7250	$InBr_3$	-5999.0293
NaCl	-1284.7232	NaBr	-3812.1191
KCl	-1310.4546	KBr	-3837.8909

Table S3. Refined structural and microstructural parameters from high-resolution synchrotron X-ray TS data of $Cs_2(Ag,Na)InCl_6$ and Cs_2KInCl_6. The microstrain parameter, $\varepsilon = \frac{\Delta d}{d} = \frac{\beta_L}{4\tan\theta}$, describes peak broadening with a $\tan\theta$ dependence,[11] where β_L is the full width at half maximum of a Lorentzian function optimized against the experimental data. The site occupancy factor (s.o.f.) of Na^+ represents its fractional occupancy within the unit cell, while B is the isotropic atomic displacement parameter. The Goodness of Fit (GoF) of each Rietveld fit is calculated as $GoF = \chi^2 = \sqrt{\frac{\sum w_m (Y_{o,m} - Y_{c,m})^2}{M-P}}$, where $w_m = \frac{1}{\sigma(Y_{o,m})^2}$ are the weight given to each data point m, $Y_{o,m}$ and $Y_{c,m}$ are the observed and calculated data (respectively) at a data point m, M the number of data point and P the number of parameters of the model. The refined atomic coordinates are provided in the *cif files deposited alongside the Supporting Information file.

Sample	Unit cell parameter (Å)	ε (x100)	Size (nm)	B(Cs) (Å^2)	B(In) (Å^2)	B(Cl) (Å^2)	B(Ag,Na, K) (Å^2)	s.o.f. (Ag^+)	GoF
$Cs_2AgInCl_6$	10.4858(2)	0.100(2)	37.0(5)	2.48(2)	0.27(4)	1.89(3)	2.25(6)	-	1.232
$Cs_2Na_{0.34}Ag_{0.66}InCl_6$	10.4989(2)	0.0759(9)	45.8(5)	2.84(3)	0.66(4)	2.12(4)	1.59(8)	0.664(6)	1.370
$Cs_2Na_{0.62}Ag_{0.38}InCl_6$	10.5192(3)	0.113(2)	24.6(3)	2.81(3)	0.51(3)	1.84(4)	1.7(1)	0.382(5)	1.109
$Cs_2Na_{0.82}Ag_{0.18}InCl_6$	10.5352(2)	0.099(3)	38(1)	3.65(7)	1.30(8)	2.32(8)	3.0(3)	0.182(9)	0.950
$Cs_2NaInCl_6$	10.53188(5)	0.0183(9)	87(2)	2.67(6)	0.91(6)	2.20(7)	1.7(3)	-	0.861
Cs_2KInCl_6 295K	16.97349(3), 10.99503(3)	0.0381(9)	271(2)	3.32(2)	0.81(2)	2.19(3)	2.2(1)	-	1.857
Cs_2KInCl_6 400K	10.93009(1)	0.0555(2)	186(1)	5.16(2)	1.58(1)	5.73(3)	5.10(8)	-	1.740

Note to Table S3: the average NCs size derived from the Rietveld refinement of the synchrotron X-ray TS data analysis is in good agreement with the TEM image analysis shown in Figure S1. Minor differences are expected given the increased sensitivity of X-ray diffraction-based methods to larger volume particles. The largest deviation is observed for $Cs_2NaInCl_6$, probably due to the coalescence of NCs during storage/transport. Rietveld refinement of the laboratory data of a (fresh) sample with the same composition yielded an average size of 27(4) nm, in closer agreement with the TEM value of Figure S1.

Table S4. Geometrical and electronic parameters of all DFT-optimized structures in cubic symmetry. Total energy is computed with respect to isolated atoms.

	Cubic lattice parameter (Å)	Direct bandgap (eV)	Total Energy (eV/atom)
**$Cs_2AgInCl_6$**	10.66690(1)	0.99	-2822.65
$Cs_2Na_{0.25}Ag_{0.75}InCl_6$	10.69006(1)	1.27	-2739.03
$Cs_2Na_{0.5}Ag_{0.5}InCl_6$	10.70854(1)	1.54	-2655.41
$Cs_2Na_{0.75}Ag_{0.25}InCl_6$	10.73467(1)	2.13	-2571.78
**$Cs_2NaInCl_6$**	10.76229(1)	2.92	-2486.61
$Cs_2Na_{0.75}K_{0.25}InCl_6$	10.86568(1)	3.04	-2489.44
$Cs_2Na_{0.5}K_{0.5}InCl_6$	10.97753(1)	3.15	-2490.73
$Cs_2Na_{0.25}K_{0.75}InCl_6$	11.05965(1)	3.28	-2492.01
**Cs_2KInCl_6**	11.15907(1)	3.42	-2493.30

Table S5. Geometrical and electronic parameters of Cs_2KInCl_6 in different space group symmetries, optimized with DFT. Total energy is computed with respect to isolated atoms.

**Space group**	**Lattice parameters (Å)**	**Direct Bandgap (eV)**	**Total Energy (eV/atom)**
Fm-3m	*a* = *b* = *c* = 11.15907(1)	3.42	-2493.3009
P4/mnc (*tetragonal* in Fig. 4)	*a* = *b* = 7.87488(1)<br>*c* = 11.18217(1)	3.44	-2493.3017
I4/m (*tetragonal* in Fig. 4)	*a* = *b* = 7.81776(1)<br>*c* = 11.05599(1)	3.47	-2493.3051
I4/m (*distorted tetragonal* in Fig. 4)	*a* = *b* = 7.81776(1)<br>*c* = 11.24222(1)	3.35	-2493.3058

7. Supplementary Figures

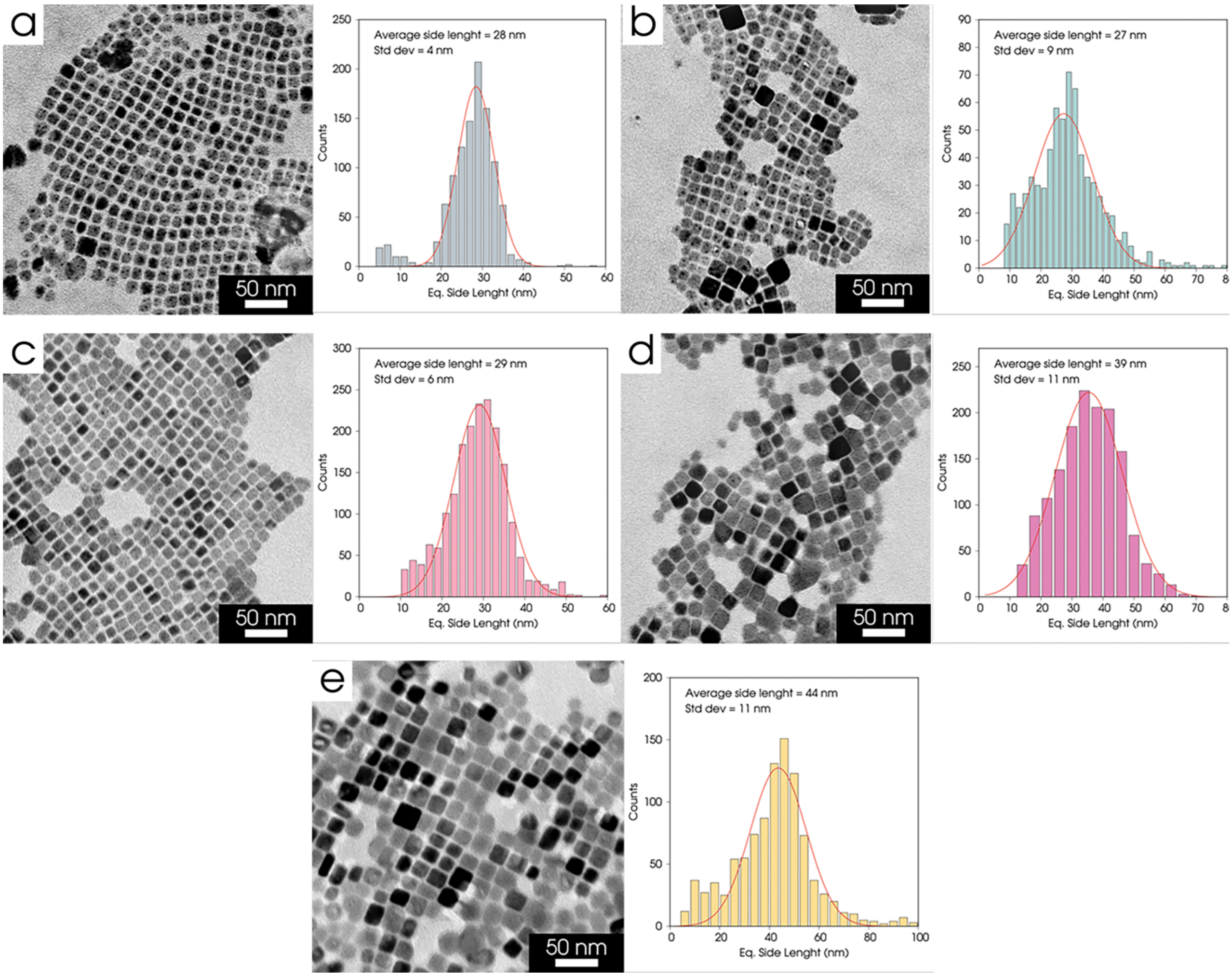

Figure S1. TEM images of $Cs_2(Na,Ag)InCl_6$ NCs, with a scale bar of 50 nm, and corresponding size distribution histograms: (a) $Cs_2AgInCl_6$, (b) $Cs_2Na_{0.34}Ag_{0.66}InCl_6$, (c) $Cs_2Na_{0.62}Ag_{0.38}InCl_6$, (d) $Cs_2Na_{0.82}Ag_{0.18}InCl_6$, and (e) $Cs_2NaInCl_6$.

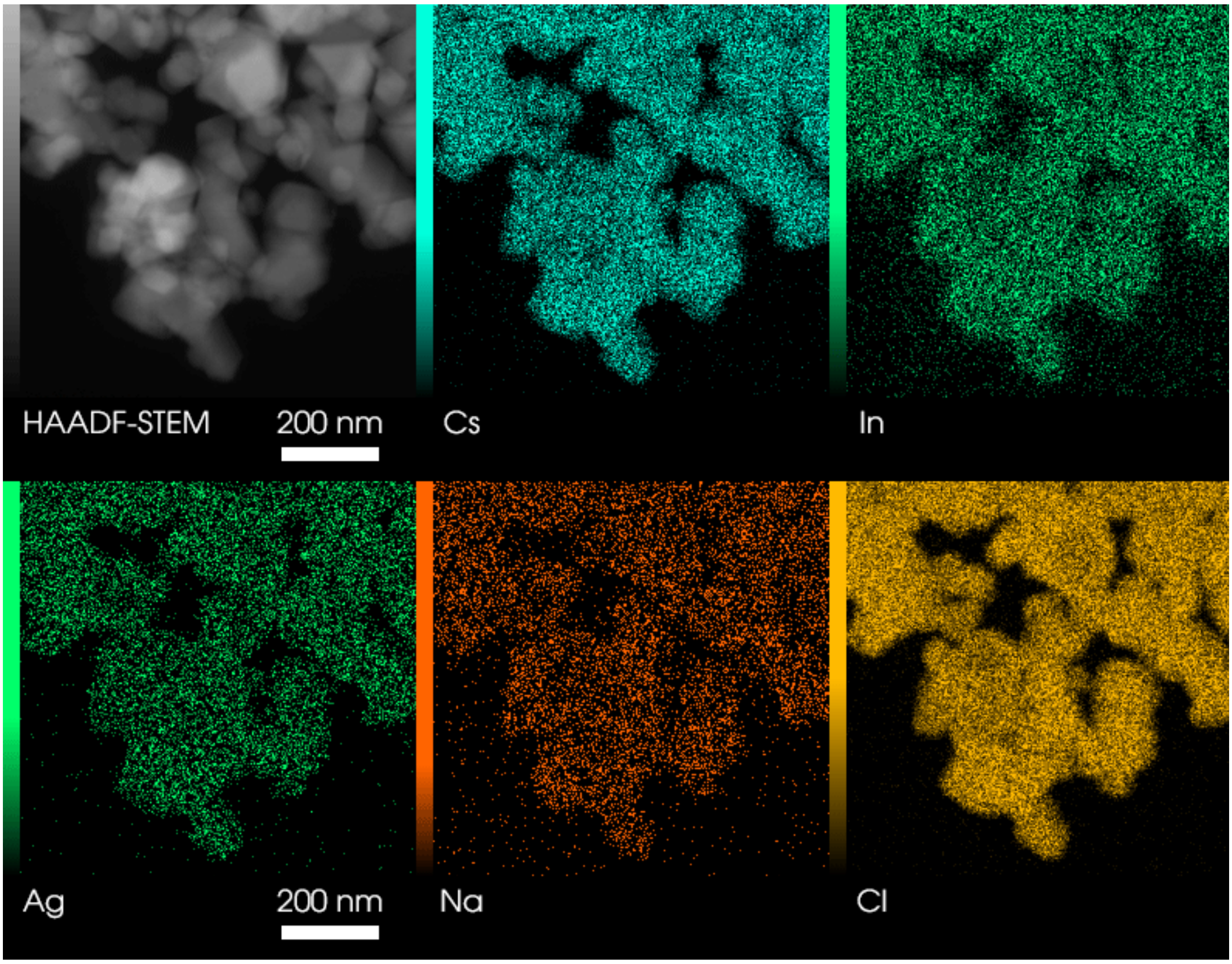

Figure S2. High-angle annular dark-field (HAADF) scanning transmission electron microscopy (HAADF-STEM) of the $Cs_2Na_{0.62}Ag_{0.38}InCl_6$ sample and energy-dispersive X-ray spectroscopy (EDX) mapping of the same area for the elements Cs, Na, Ag, In, and Cl.

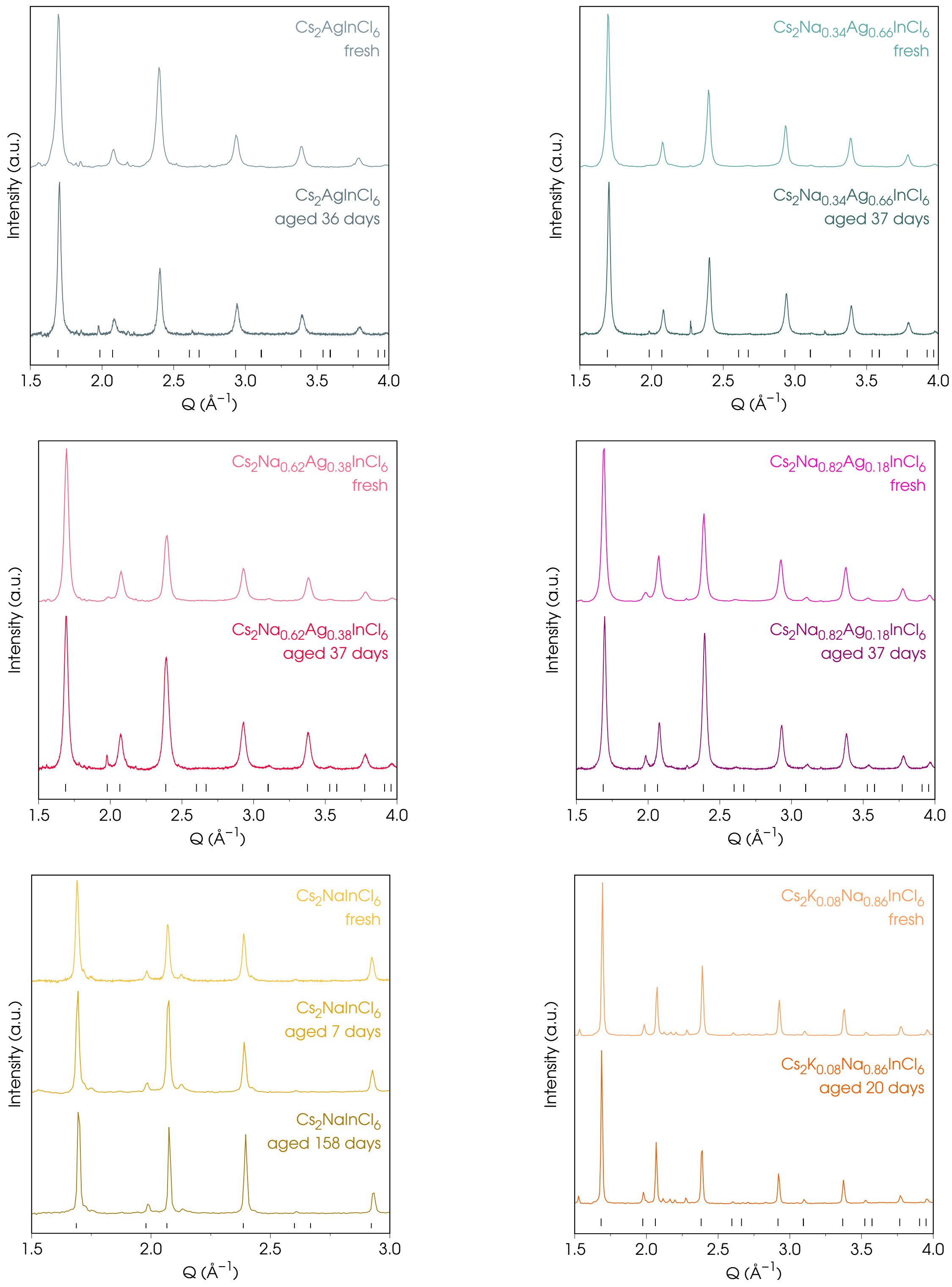

$Cs_2AgInCl_6$
fresh
$Cs_2AgInCl_6$
aged 36 days
$Cs_2Na_{0.34}Ag_{0.66}InCl_6$
fresh
$Cs_2Na_{0.34}Ag_{0.66}InCl_6$
aged 37 days
$Cs_2Na_{0.62}Ag_{0.38}InCl_6$
fresh
$Cs_2Na_{0.62}Ag_{0.38}InCl_6$
aged 37 days
$Cs_2Na_{0.82}Ag_{0.18}InCl_6$
fresh
$Cs_2Na_{0.82}Ag_{0.18}InCl_6$
aged 37 days
$Cs_2NaInCl_6$
fresh
$Cs_2NaInCl_6$
aged 7 days
$Cs_2NaInCl_6$
aged 158 days
$Cs_2K_{0.08}Na_{0.86}InCl_6$
fresh
$Cs_2K_{0.08}Na_{0.86}InCl_6$
aged 20 days
Intensity (a.u.)
Q ($Å^{-1}$)
1.5
2.0
2.5
3.0
3.5
4.0

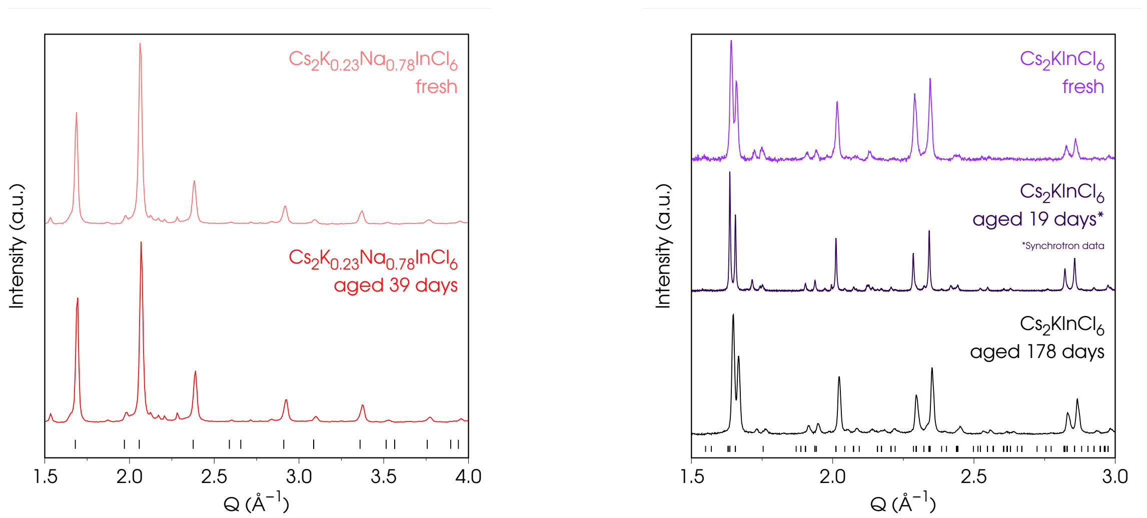

Figure S3. Laboratory X-ray powder diffraction data on freshly prepared and aged $Cs_2(Na,Ag)InCl_6$ and $Cs_2(Na,K)InCl_6$ NCs, measured in flat plate geometry after drying on a zero background sample holder. The changes in the intensity ratios of the main Bragg peaks, compared to Figure 2 and 4 of the main text, are attributed to preferred orientation effects, which are commonly observed in faceted NCs lying on a flat surface.

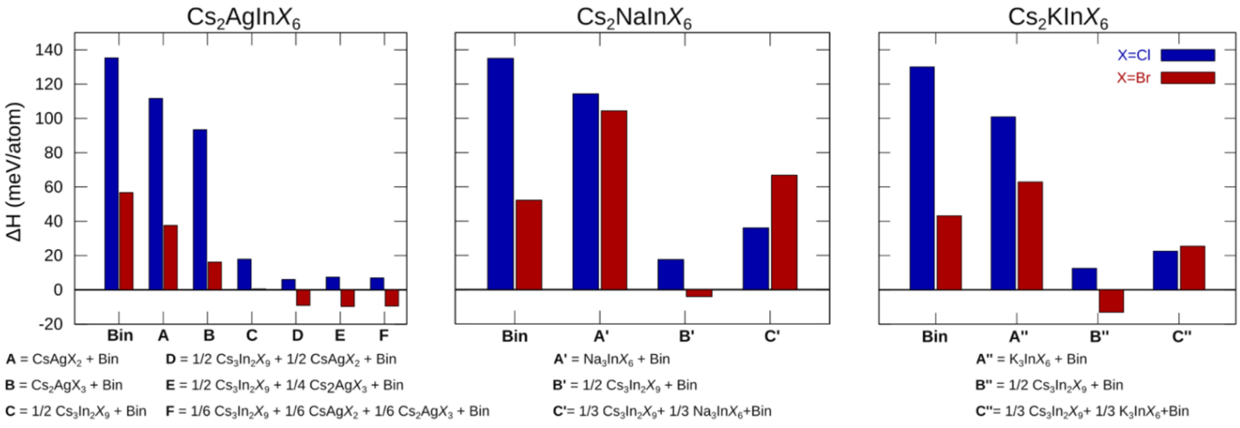

Figure S4. Reaction enthalpy for the decomposition to different products, calculated with DFT for Cs_2AgInX_6, Cs_2NaInX_6 and Cs_2KInX_6. Enthalpies for chlorides and bromides are shown as blue and red bars, respectively.

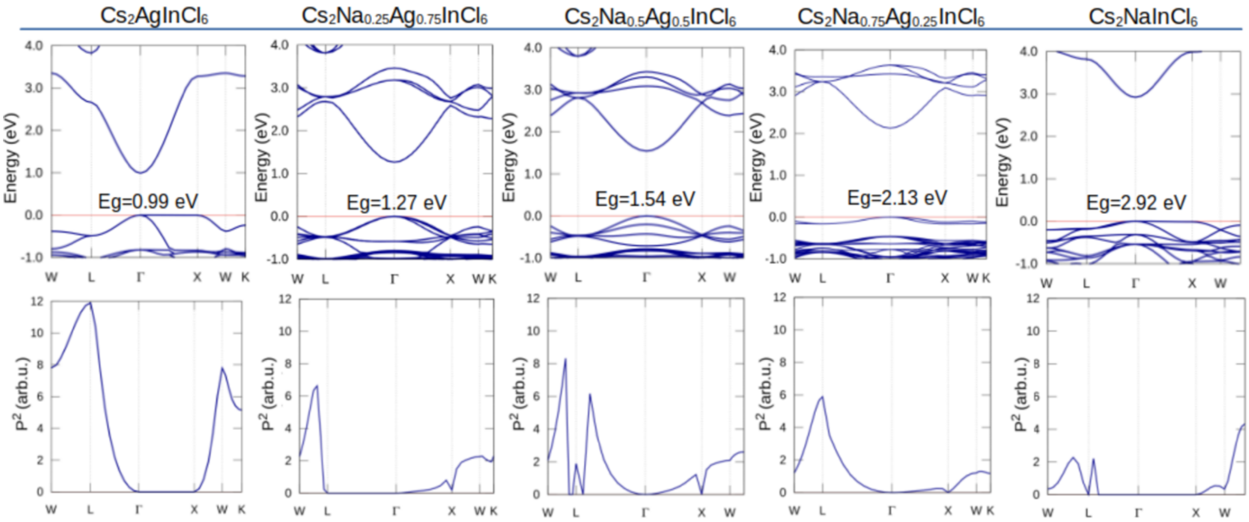

Figure S5. Band structure and dipole transition elements of $Cs_2(Na,Ag)InCl_6$.

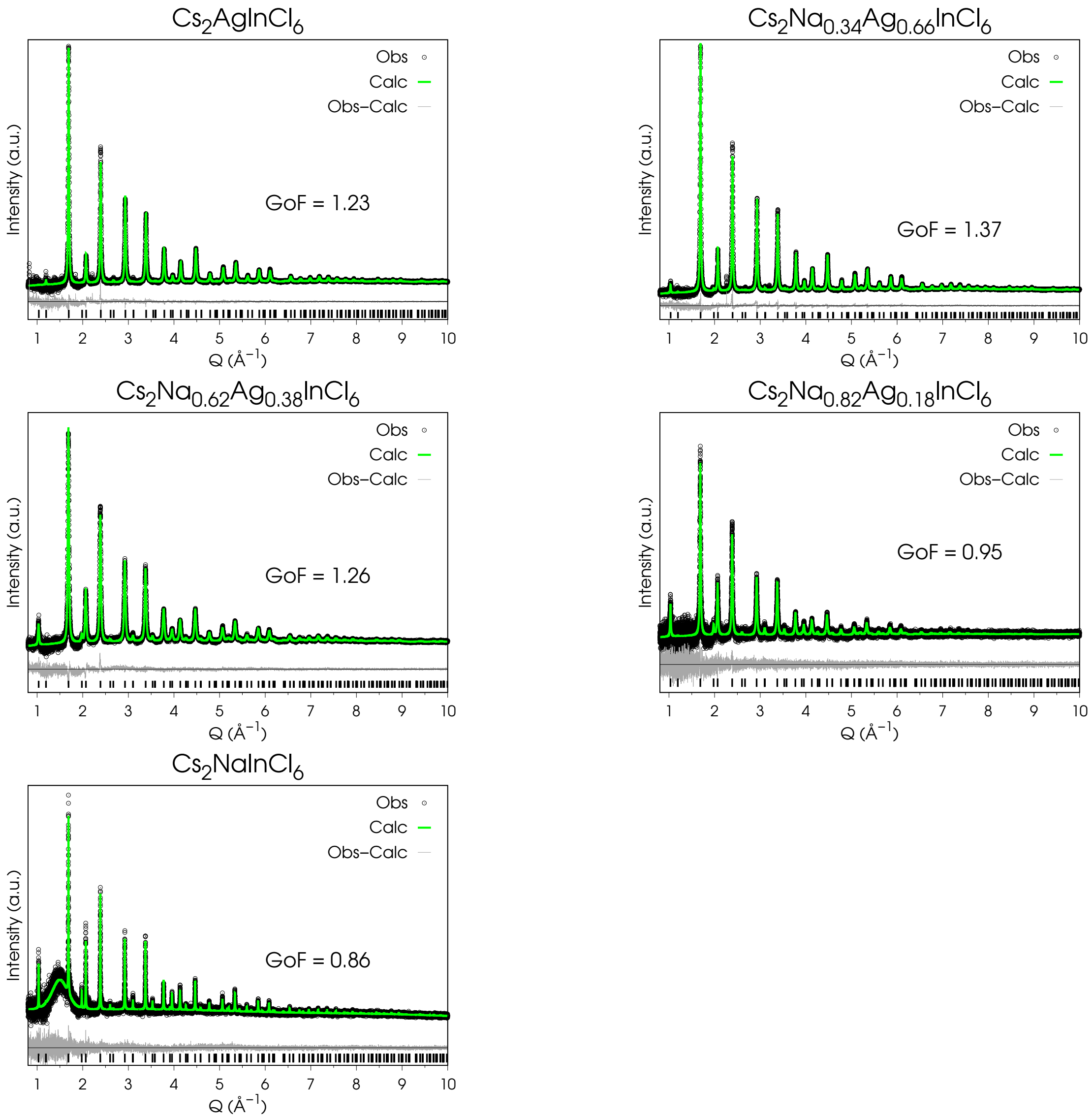

Figure S6. Best fits of RT synchrotron WAXTS of $Cs_2(Na,Ag)InCl_6$ colloidal NCs (with the toluene blank trace subtracted): experimental data (black dots), calculated pattern (green traces), residual trace (grey trace). The corresponding refined structural parameters are available in Table S3.

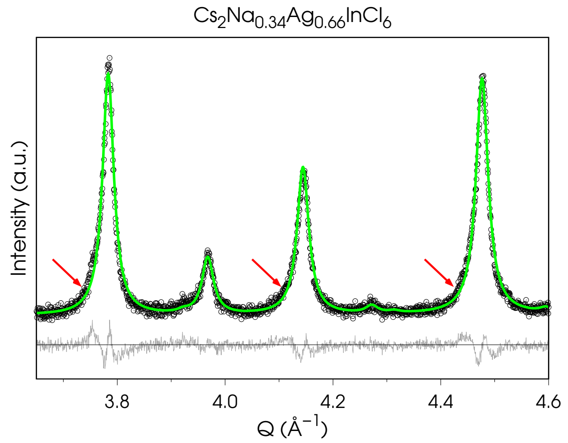

Figure S7. Zoomed-in view of the best fit of high-resolution synchrotron WAXTS data for $Cs_2Na_{0.34}Ag_{0.66}InCl_6$ colloidal NCs (with the toluene blank trace subtracted). Experimental data are shown as black dots, the calculated pattern as green traces, and the residual trace as a grey line. Red arrows highlight the broad low-angle shoulders present in all reflections of the WAXTS pattern, indicating deviations from an ideal solid solution.

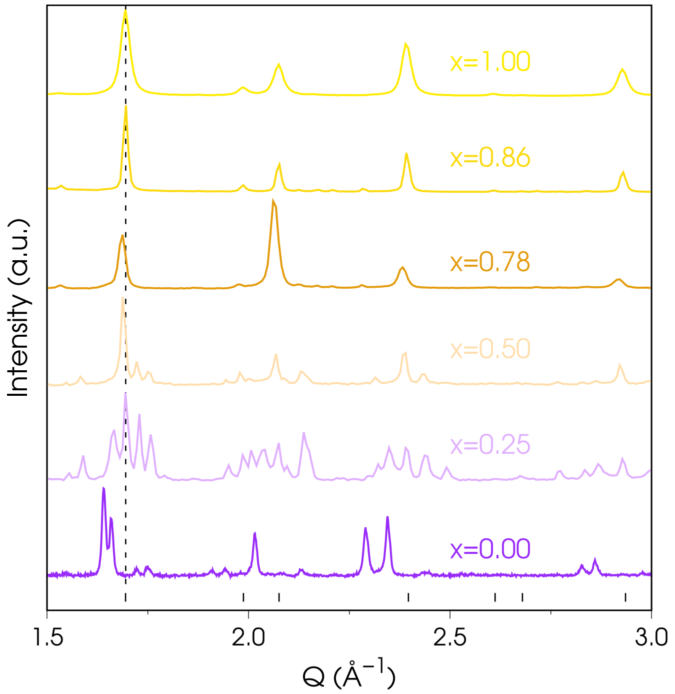

Figure S8. Laboratory X-ray diffraction data for $Cs_2Na_xK_{1-x}InCl_6$ NCs (with $0 \leq x \leq 1.0$), indicating the limited miscibility of K^+ within the 3D HDP *elpasolite* $Cs_2NaInCl_6$ structure (black tics at the bottom of the plot). For $x(Na^+) < 0.78$ the precipitation of a hydrated 0D perovskite phase[25] is observed together with the pristine Na-rich 3D perovskite. The compositions reported in the figure for $0.78 \leq x(Na^+) \leq 0.25$ correspond to nominal values rather than the actual Na^+ fraction incorporated into the perovskite framework.

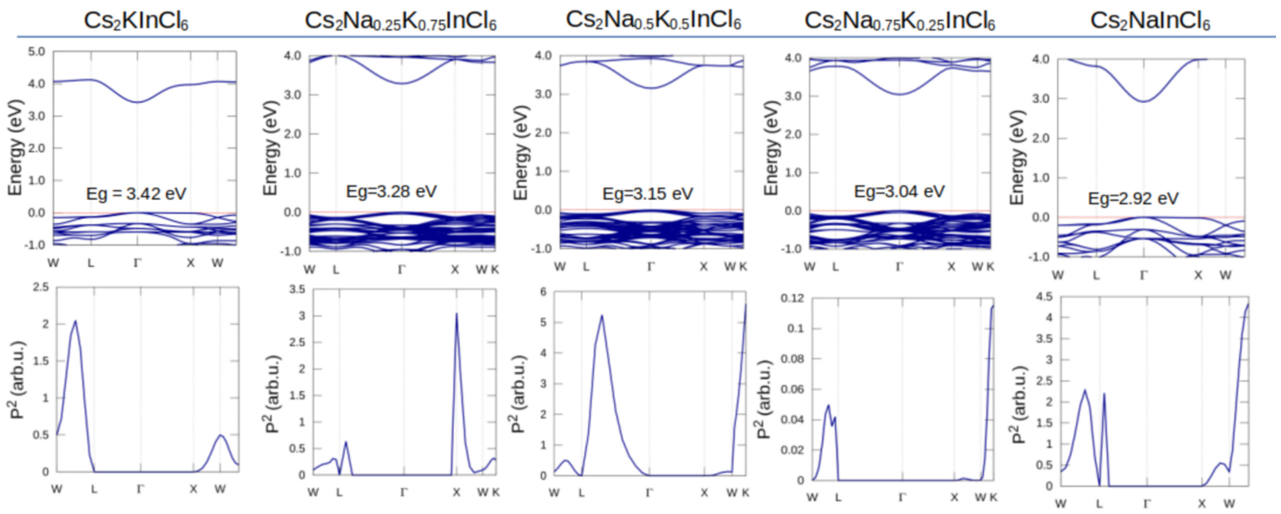

Figure S9. Band structure and dipole transition elements of $Cs_2(Na,K)InCl_6$

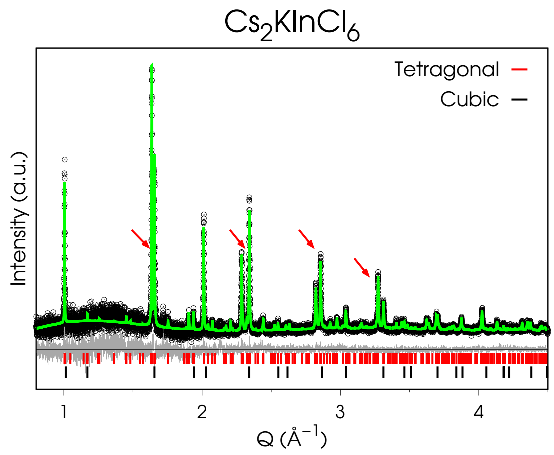

Figure S10. Zoomed-in view of the best fits of high-resolution WAXTS data of Cs_2KInCl_6 colloidal NCs in toluene, with red arrows highlighting the systematic splitting of the main reflections, indicating a deviation from the face-centered cubic *elpasolite* crystal structure. The corresponding refined structural parameters of the distorted *I4/m* tetragonal structure (further confirmed on dry data, Figure S11) are available in Table S3.

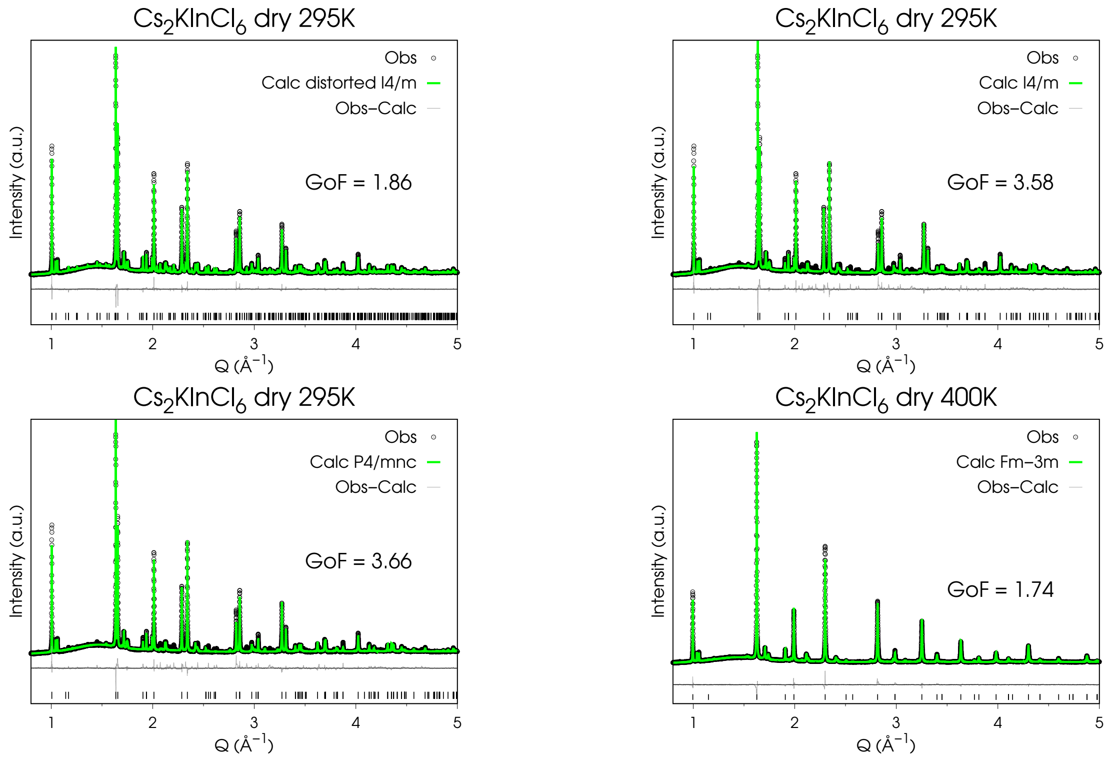

Figure S11. Zoomed-in view of the best fits of (RT and high temperature) synchrotron WAXTS data of Cs_2KInCl_6 dry NCs obtained with three tetragonal phases (distorted *I4/m* and the two "regular" *I4/m* and *P4/mnc* structures): experimental data (black dots), calculated pattern (green traces), residual trace (grey trace). The corresponding refined structural parameters are available in Table S3.

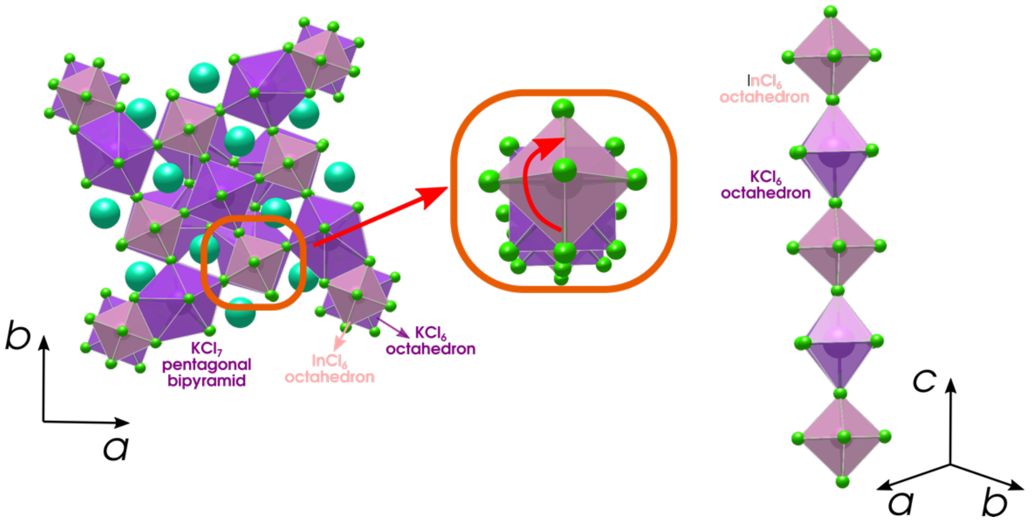

Figure S12. Atomistic model of Cs_2KInCl_6 *I4/m* crystal structure refined from RT data. **Left:** View along the [001] direction (*ab*-plane of the tetragonal unit cell), showing the different coordination polyhedra. The inset highlights a twisted chain of $(K,In)Cl_6$ octahedra running parallel to the unit cell *c*-axis. **Right**: view along [110] of a single $(K,In)Cl_6$ twisted chain oriented along the *c*-axis.

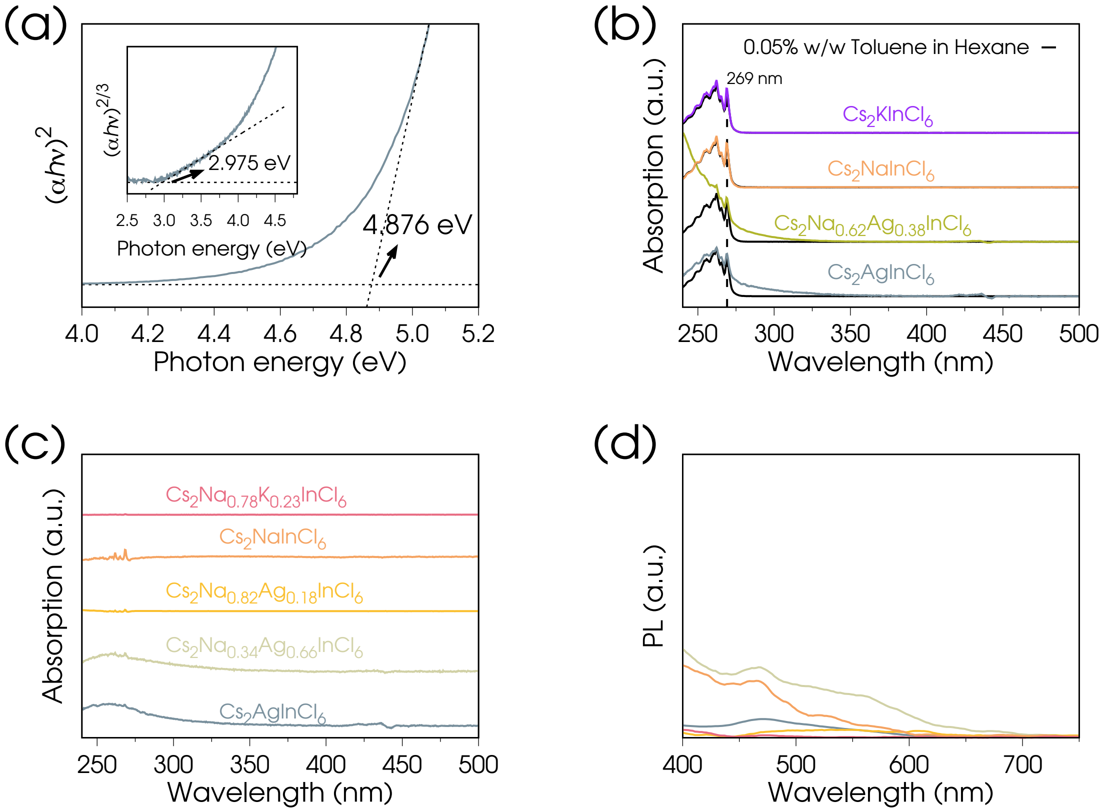

Figure S13. (a) Tauc plot of $Cs_2Ag_{0.62}In_{0.38}Cl_6$ NCs obtained by fitting the strong absorption tail in the 5.0 - 5.2 eV energy range. Inset: zoomed-in view of the lowest-energy absorption feature and the corresponding Tauc fit performed in the 3.2 -3.7 eV range. (b) UV-Vis absorption spectra of selected $Cs_2B^+InCl_6$ compositions showing the residual toluene contributions with the sharp vibronic peak at 269 nm (the corresponding "clean" spectra in hexane after vacuum drying and complete toluene removal are shown in Figure 5 of the main text). (c) UV-Vis absorption spectra of selected $Cs_2B^+InCl_6$ compositions after subtraction of the 0.05% w/w toluene in hexane trace shown in (b) and (d) the corresponding photoluminescence spectra using the same color codes; the other NCs compositions are shown in Figure 5 of the main text.

(1) Anker, A. S.; Jensen, J. H.; Gonzalez-Duque, M.; Moreno, R.; Smolska, A.; Juelsholt, M.; Hardion, V.; Jorgensen, M. R. V.; Faina, A.; Quinson, J.; Stoy, K.; Vegge, T. Autonomous Nanoparticle Synthesis by Design. arXiv May 19, 2025. http://arxiv.org/abs/2505.13571 (accessed 2025-06-29).
(2) Epps, R. W.; Bowen, M. S.; Volk, A. A.; Abdel-Latif, K.; Han, S.; Reyes, K. G.; Amassian, A.; Abolhasani, M. Artificial Chemist: An Autonomous Quantum Dot Synthesis Bot. *Advanced Materials* **2020**, *32*, 2001626.
(3) Burger, B.; Maffettone, P. M.; Gusev, V. V.; Aitchison, C. M.; Bai, Y.; Wang, X.; Li, X.; Alston, B. M.; Li, B.; Clowes, R.; Rankin, N.; Harris, B.; Sprick, R. S.; Cooper, A. I. A Mobile Robotic Chemist. *Nature* **2020**, *583*, 237–241.
(4) Salley, D.; Manzano, J. S.; Kitson, P. J.; Cronin, L. Robotic Modules for the Programmable Chemputation of Molecules and Materials. *ACS Cent. Sci.* **2023**, *9*, 1525–1537.
(5) Fitch, A.; Dejoie, C.; Covacci, E.; Confalonieri, G.; Grendal, O.; Claustre, L.; Guillou, P.; Kieffer, J.; Nolf, W. de; Petitdemange, S.; Ruat, M.; Watier, Y. ID22 – the High-Resolution Powder-Diffraction Beamline at ESRF. *J Synchrotron Rad* **2023**, *30*, 1003–1012.
(6) Paalman, H. H.; Pings, C. J. Numerical Evaluation of X-Ray Absorption Factors for Cylindrical Samples and Annular Sample Cells. *J. Appl. Phys.* **1962**, *33*, 2635–2639.
(7) Bowden, M.; Ryan, M. Absorption Correction for Cylindrical and Annular Specimens and Their Containers or Supports. *J. Appl. Crystallogr.* **2010**, *43*, 693–698.
(8) Topas-R, V3.0, 2005.
(9) McCall, K. M.; Friedrich, D.; Chica, D. G.; Cai, W.; Stoumpos, C. C.; Alexander, G. C. B.; Deemyad, S.; Wessels, B. W.; Kanatzidis, M. G. Perovskites with a Twist: Strong In^{1+} Off-Centering in the Mixed-Valent $CsInX_3$ (X = Cl, Br). *Chem. Mater.* **2019**, *31*, 9554–9566.
(10) Howard, C. J.; Kennedy, B. J.; Woodward, P. M. Ordered Double Perovskites – a Group-Theoretical Analysis. *Acta Cryst B* **2003**, *59*, 463–471.
(11) Balzar, D. Voigt Function Model in Diffraction-Line Broadening Analysis. In *Defect and Microstructure Analysis by Diffraction*; Snyder, R. L., Fiala, J., Bunge, H. J., Eds.; Oxford University Press, 2000; pp 94–126.
(12) Dal Corso, A. Pseudopotentials Periodic Table: From H to Pu. *Computational Materials Science* **2014**, *95*, 337–350.
(13) Xiao, Z.; Du, K.-Z.; Meng, W.; Mitzi, D. B.; Yan, Y. Chemical Origin of the Stability Difference between Copper(I)- and Silver(I)-Based Halide Double Perovskites. *Angew. Chem. Int. Ed. Engl.* **2017**, *56*, 12107–12111.
(14) Kale, A. J.; Chaurasiya, R.; Dixit, A. Lead-Free $Cs_2BB'X_6$ (B: Ag/Au/Cu, B′: Bi/Sb/Tl, and X: Br/Cl/I) Double Perovskites and Their Potential in Energy Conversion Applications. *ACS Appl. Energy Mater.* **2022**, *5*, 10427–10445.
(15) Bartel, C. J.; Clary, J. M.; Sutton, C.; Vigil-Fowler, D.; Goldsmith, B. R.; Holder, A. M.; Musgrave, C. B. Inorganic Halide Double Perovskites with Optoelectronic Properties Modulated by Sublattice Mixing. *J. Am. Chem. Soc.* **2020**, *142*, 5135–5145.
(16) Han, S.; Tu, D.; Xie, Z.; Zhang, Y.; Li, J.; Pei, Y.; Xu, J.; Gong, Z.; Chen, X. Unveiling Local Electronic Structure of Lanthanide-Doped Cs2NaInCl6 Double Perovskites for Realizing Efficient Near-Infrared Luminescence. *Advanced Science* **2022**, *9*, 2203735.
(17) Zhou, J.; Rong, X.; Zhang, P.; Molokeev, M. S.; Wei, P.; Liu, Q.; Zhang, X.; Xia, Z. Manipulation of Bi^{3+}/In^{3+} Transmutation and Mn^{2+}-Doping Effect on the Structure and Optical Properties of Double Perovskite $Cs_2NaBi_{1-x}In_xCl_6$. *Adv. Opt. Mater.* **2019**, *7*, 1801435.
(18) Zeng, R.; Zhang, L.; Xue, Y.; Ke, B.; Zhao, Z.; Huang, D.; Wei, Q.; Zhou, W.; Zou, B. Highly Efficient Blue Emission from Self-Trapped Excitons in Stable Sb^{3+}-Doped $Cs_2NaInCl_6$ Double Perovskites. *J. Phys. Chem. Lett.* **2020**, *11*, 2053–2061.
(19) Huang, W.; Peng, H.; Wei, Q.; Xia, J.; He, X.; Ke, B.; Tian, Y.; Zou, B. Tunable Efficient

White Emission in Holmium Doped Double Perovskites Cs2KInCl6 via Antimony Sensitization. *Advanced Optical Materials* **2023**, *11*, 2203103.
(20) Luo, J.; Wang, X.; Li, S.; Liu, J.; Guo, Y.; Niu, G.; Yao, L.; Fu, Y.; Gao, L.; Dong, Q.; Zhao, C.; Leng, M.; Ma, F.; Liang, W.; Wang, L.; Jin, S.; Han, J.; Zhang, L.; Etheridge, J.; Wang, J.; Yan, Y.; Sargent, E. H.; Tang, J. Efficient and Stable Emission of Warm-White Light from Lead-Free Halide Double Perovskites. *Nature* **2018**, *563*, 541–545.
(21) Locardi, F.; Sartori, E.; Buha, J.; Zito, J.; Prato, M.; Pinchetti, V.; Zaffalon, M. L.; Ferretti, M.; Brovelli, S.; Infante, I.; De Trizio, L.; Manna, L. Emissive Bi-Doped Double Perovskite $Cs_2Ag_{1-x}Na_xInCl_6$ Nanocrystals. *ACS Energy Lett.* **2019**, *4*, 1976–1982.
(22) Horne, D. S. Determination of the Fractal Dimension Using Turbidimetric Techniques. Application to Aggregating Protein Systems. *Faraday Discuss. Chem. Soc.* **1987**, *83*, 259–270.
(23) Anzini, P.; Redoglio, D.; Rocco, M.; Masciocchi, N.; Ferri, F. Light Scattering and Turbidimetry Techniques for the Characterization of Nanoparticles and Nanostructured Networks. *Nanomater.* **2022**, *12*, 2214.
(24) Tauc, J.; Grigorovici, R.; Vancu, A. Optical Properties and Electronic Structure of Amorphous Germanium. *Phys. Status Solidi B* **1966**, *15*, 627–637.
(25) Noculak, A.; Morad, V.; McCall, K. M.; Yakunin, S.; Shynkarenko, Y.; Wörle, M.; Kovalenko, M. V. Bright Blue and Green Luminescence of Sb(III) in Double Perovskite Cs_2MInCl_6 (M = Na, K) Matrices. *Chem. Mater.* **2020**, *32*, 5118–5124.
